# Broad Applications of Distributed Lag Non‐Linear Model in Public Health: A Comprehensive Review

**DOI:** 10.1029/2025GH001608

**Published:** 2025-12-09

**Authors:** Ambreen Shafqat, Eunsik Park

**Affiliations:** ^1^ Department of Mathematics and Statistics Chonnam National University Gwangju South Korea

**Keywords:** public health outcomes, distributed lag non‐linear model (DLNM), environmental exposure, time series analysis, exposure‐response relationships

## Abstract

This study reviews the use of the distributed lag non‐linear model (DLNM) in public health research, focusing on environmental‐exposure, health–outcome relationships, and providing recommendations for future studies. Embase, PubMed, Web of Science, and Scopus databases were searched for literature published from January 2020 to November 2024 using the DLNM to analyze the environmental exposures and health outcomes. After screening, removing duplicates, and reviewing full‐text articles, eligible studies were assessed using the DLNM to examine the health effects related to environmental exposure, particularly temperature and other environmental factors. From 2,847 studies, 274 studies from 36 countries were selected for analysis, primarily from China (164), Europe (28), and North America (23). There were 174 exclusive climate data sources, no standardized heat thresholds, and 131 unique sources of air pollutant data. Among the 53 adverse health outcomes identified using the DLNM, morbidity was the most prevalent (n=102), followed by hospitalization (n=39), hospital admission (n=40), and emergency room visits (n=22). This review highlights the utility of the DLNM in capturing complex temporal relationships between environmental exposure and health, clarifying lagged effects. Despite the challenges of standardization and computational efficiency, ongoing developments are enhancing the utility of the DLNM. Future research should focus on advanced statistical techniques, such as machine learning and neural networks, and extend applications to other environmental health scenarios.

## Plain Language Summary

The study reviews the use of distributed lag non‐linear models (DLNM) in public health research, particularly in examining the relationships between environmental exposures and health outcomes. It analyzed 274 studies from various countries, primarily China, Europe, and North America, focusing on health effects related to environmental factors like temperature and air pollutants. The review found that morbidity was the most common adverse health outcome identified. It emphasizes the importance of DLNM in understanding the complex and delayed effects of environmental exposures on health. The study suggests that future research should explore advanced statistical methods, including machine learning, to further enhance the application of DLNM in environmental health.

## Key Points

- This study reviews the use of DLNM to analyze complex, lagged health effects from environmental exposures like temperature and air pollution
- Most DLNM studies focus on CVDs, RDs, and infectious diseases, with limited applications to mental, neurological, and renal health
- Integrating DLNM with Machine Learning and Bayesian models can enhance predictive accuracy and address complex multiexposure health risks

## 1 Introduction

Advanced analytical frameworks are necessary to capture the nonlinear and time‐dependent effects of the complex relationship between environmental exposure and public health. Among these frameworks, the distributed lag nonlinear model (DLNM) has emerged as a robust statistical tool that integrates exposure–response relationships with lag structures, enabling researchers to evaluate how health outcomes evolve in response to environmental changes. The functionality of the DLNM lies in its capacity to estimate the delayed and cumulative effects of exposure (e.g., temperature, air pollution, and humidity), which often interact nonlinearly with health metrics. The increasing availability of high‐resolution temporal and spatial data has promoted the widespread adoption of the DLNM in public health research. This model has been extensively employed to examine cardiovascular diseases (CVDs), respiratory diseases (RDs), and infectious diseases. However, its application remains limited in other domains (e.g., mental health and neurological disorders) and studies conducted in low‐ and middle‐income countries. As public health challenges become more complex due to climate change and urbanization, the need for advanced modeling techniques to capture multiexposure scenarios and cumulative effects has become urgent.

This review offers a comprehensive synthesis of DLNM applications across diverse environmental exposures and disease outcomes, categorizing studies by geography, exposure type, and response variable. Moreover, this review assesses the contributions of the DLNM to public health research, identifies methodological and geographical gaps, and proposes future directions for expanding its functionality through integration with machine learning (ML), Bayesian techniques, and other advanced methods. Furthermore, this work aims to provide a foundation for researchers and policymakers to apply the DLNM to improve public health.

## 2 Literature Review

The development of the DLNM marks a significant advancement in environmental epidemiology, building on earlier models addressing delayed health responses to environmental exposure. Early epidemiological approaches (e.g., moving averages and single‐lag models of air pollutants) are limited in capturing the complexity of non‐linear exposure–response relationships (Samoli et al., 2005). These models often oversimplify temporal dynamics, inadequately representing exposure effects over time. In light of these limitations, the DLNM was presented as a more adaptable and all‐encompassing framework to represent intricate time‐dependent and non‐linear relationships (Gasparrinia et al., 2010; Roberts & Martin, 2007). This innovation enables more precise assessments of how environmental exposures evolve to affect human health.

Since its creation, the DLNM has emerged as a critical tool for studying the effects of environmental exposure on health. For instance, Gasparrini and Armstrong (2011) demonstrated the capability of the DLNM to clarify the immediate and delayed effects of temperature fluctuations on health, thereby advancing our understanding of this effect beyond static exposure assessments. A worldwide study conducted by Lo et al. (2023) highlighted the regional differences in ideal heat measurements, demonstrating that no single temperature metric adequately reflects heat‐related hazards globally.

Furthermore, the DLNM is not limited to use for temperature effects. This model has also been applied to analyze the combined effects of temperature and air pollutants, including particulate matter (e.g., PM_2.5_), nitrogen dioxide (NO_2_), and ozone (O_3_), on public health diseases (e.g., respiratory, cardiovascular, and other diseases). Interactional effects between various environmental exposures and public health problems have been demonstrated using this integrative technique (An et al., 2025; Cheng et al., 2019; Jin et al., 2023; Xing et al., 2022; Xu et al., 2022). The effectiveness of the DLNM has been reinforced by regional studies that have estimated the effects of extreme heat events, diurnal temperature fluctuation, and air quality on hospital admissions and death rates in North America and Asia (Dong et al., 2020; Guo, Huang, et al., 2021; Islam et al., 2017; Vicedo‐Cabrera et al., 2019; Yin et al., 2019). For example, Liu et al. (2019) used the DLNM in Germany to measure the risks of acute and lagged cardiovascular mortality related to heatwaves, revealing how useful the DLNM is at capturing intricate temporal patterns in practical settings.

Recently, the DLNM has increasingly been used to model infectious disease outcomes. A study employed the DLNM in a Bayesian hierarchical framework to evaluate the effects of the standardized precipitation index and minimum temperature on dengue fever risk, finding distinct lag structures for drought and rainfall (Lowe et al., 2018). This model achieved 86% accuracy in outbreak prediction, outperforming conventional surveillance approaches. Another study investigated the effects of climate and environmental factors on hand, foot, and mouth disease (HFMD) using a dynamic model of the DLNM, finding that the risk of transmission during winter vacations was higher than during summer vacations (Tan et al., 2023). In late 2019, Wuhan, China, experienced the emergence of the coronavirus disease 2019 (COVID‐19) outbreak. At that time, researchers examined the linear correlation, which was not significant. However, the non‐linear correlations were significant due to developing an adapted‐susceptible‐exposed‐infection‐recovered model called the DLNM to analyze the relationship and lag effects between the daily new cases of COVID‐19 and meteorological factors worldwide (Fu et al., 2021; Zhang et al., 2024).

Furthermore, maternal and neonatal health have also been a focus of DLNM applications. For instance, some studies have assessed the influence of air pollutant exposure during pregnancy on birth outcomes using the DLNM to model the exposure–response relationship over the gestational weeks (Bravo et al., 2024; Wu et al., 2018). This study provides evidence that maternal avoidance of very high pollution levels of PM_2.5_ during the 27th to 33rd gestational weeks may reduce birth weight. Another study examined the link between short‐term ambient air pollution and upper gastrointestinal bleeding leading to emergency room attendance in Hong Kong (2017–2022) and analyzed 31,577 records using the DLNM, finding that exposure to high levels of PM_2.5_ significantly increased the risk of upper gastrointestinal bleeding from lag Day 3 (Li et al., 2024). These studies underscore the relevance of the DLNM in understanding critical life stages that are vulnerable to environmental risks.

The results of DLNM applications have been extended to mental and neurological disorders in recent studies. A Korean study highlighted the potential of the DLNM in analyzing the strongest relationship between mental illnesses and extremely hot temperatures within a lag period of 0–4 days (Lee et al., 2018), revealing that 14.6% of emergency admissions for mental illnesses were due to extreme hot temperatures. Another study employing a case‐crossover design with the DLNM was conducted to evaluate the complex and time‐delayed effects of temperature on five psychiatric conditions, including affective disorders, anxiety, depressive disorders, schizophrenia, and organic mental disorders (Zhang et al., 2020). They observed that low temperatures had a significant and prolonged effect on most types of mental disorders in Zhaoqing, Shenzhen, and Huizhou, China. Another study (Kim et al., 2024) clearly explained the long‐term relationship between ambient temperature and suicide risk using a time‐stratified case‐crossover design to analyze the nonlinear and time‐delayed effects. They provided detailed evidence that abnormally elevated temperatures, both anomalous and out of season, may trigger suicidal behavior, including suicidal deaths and intentional self‐harm.

Moreover, DLNM research has been focused on economically advanced regions, primarily in North America (31.8%) and China (68.9%) because of the availability of substantial environmental and health data resources (Graffy et al., 2024). Most studies have relied on time‐series and case‐crossover designs, which model acute effects well but often fail to address long‐term cumulative exposures. Although longitudinal frameworks and Bayesian extensions exist, their use in DLNM studies remains rare (Gao et al., 2023; Gasparrini & Armstrong, 2010; Gasparrini et al., 2017; Kim et al., 2021; Luo et al., 2022; Vicedo Cabrera et al., 2020; Wilson et al., 2017, 2022).

Additionally, the management of seasonality and exposure standardization presents further methodological difficulties that cannot be managed using DLNM techniques. Furthermore, the DLNM has rarely been integrated with ML, deep learning, or spatial modeling approaches that could enhance prediction, uncertainty estimation, and cross‐regional generalization. These limitations underscore a significant gap in the literature regarding the convergence of the DLNM with innovative analytical methods.

Although prior literature has addressed the use of the DLNM regarding specific diseases or regions, a comprehensive synthesis of its broad applications across public health domains remains necessary. This review aims to fill this gap by providing a cross‐sectional analysis of DLNM studies categorized by disease type, exposure variable, geographic scope, and modeling strategy. This analysis evaluates the methodological strengths and limitations of the DLNM, identifies emerging applications, and outlines critical opportunities for interdisciplinary integration and future development.

## 3 Method and Materials

### 3.1 Search Schemes and Inclusion/Exclusion Criteria

We conducted and reported extensive evaluations according to the Preferred Reporting Items for Systematic reviews and Meta‐Analyses guidelines to maintain transparency and guide the review process (Colquhoun et al., 2014; Munn et al., 2018; Tsui et al., 2008; Table 1). We conducted the initial systematic exploration from January 2020 to November 2024 using four databases (PubMed, Web of Science, Scopus, and the Cochrane Library). The keywords and search strategies for these databases were “distributed lag nonlinear models” OR “DLNM.” In addition, we manually checked the references of all included studies.

**Table 1:** Inclusion and Exclusion Criteria

Inclusion criteria	Exclusion criteria
Studies that applied the DLNM in public health	Studies that do not link the DLNM to public health effects
Studies that examined the link between environmental exposure (e.g., temperature, air pollution, and metrology) and public health effects	Studies that link various exposures, such as hot baths, saunas, and experimental temperatures
Studies that focus on health‐related outcomes	Studies that did not assess public health consequences
Original research studies that were published in English	Articles that were not published in English
Various study designs	Reviews of gray literature
Articles that were published during the period from January 2020 to November 2024	Articles that were published before or after the collection period

### 3.2 Data Extraction

**Figure 1:**
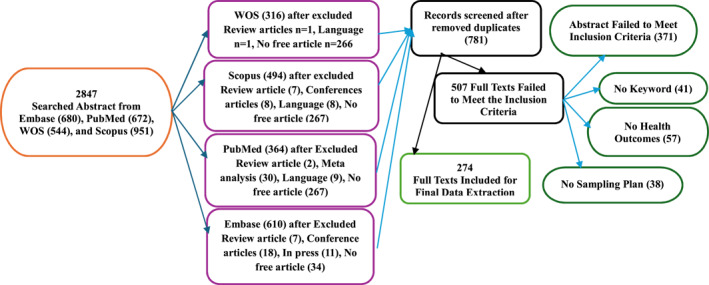
Flow chart of the abstract and full‐text inclusion and exclusion criteria.

We employed a combination of keyword searches and subject headings focused on environmental exposure, statistical modeling approaches, and associated health outcomes to identify relevant studies. Afterward, we imported all identified articles into an Excel spreadsheet and removed duplicates using R software (v. 4.4.2). We conducted this selection process in two stages. First, we screened the titles and abstracts to filter out irrelevant studies. Next, we conducted a full‐text review of the remaining studies based on the defined inclusion and exclusion criteria. We employed the R program to enhance the specialized data abstraction program created specifically for this research to analyze the titles and abstracts of the studies. This custom program streamlined the process of extracting crucial information on study characteristics, including methodologies, exposure definitions, and primary outcomes. During the full‐text data extraction phase, various variables were recorded, including the publication year, country of study, effect size, exposure variables, associated diseases, response variables, sampling design, and statistical modeling techniques. Figure 1 visually summarizes the entire selection process, outlining each step of the review method.

## 4 Results

The initial analysis of the four databases yielded 2,847 articles, which we screened. After applying the preliminary search conditions to each database, 1,784 articles remained (Embase, 610; PubMed, 364; Scopus, 494; and Web of Science, 316). Among these, we identified 1,003 articles as duplicates, resulting in 781 studies for title and abstract assessment. Of these, we eliminated 507 articles, and we employed the remaining 274 articles for the preliminary data analysis (see Figure 1).

### 4.1 Sampling Designs and Health Outcomes Across Disease Categories

We examined the study designs and health outcomes in all 274 included articles to explore the methodological trends in DLNM research. While DLNM's versatility is evident across various study designs, a strong inclination toward time‐series and case‐crossover designs suggests a dominant focus on acute or short‐to‐medium term effects, particularly for morbidity outcomes. As presented in Table S1 of Supporting Information S1, most studies (n=128) employed a quantitative time‐series design (47%), followed by a case‐crossover design in 34 studies (12.5%) and a cohort design in 21 studies (7.6%). Other sampling designs (e.g., cross‐sectional and observational studies) and advanced techniques (e.g., deep learning or ML) were comparatively rare.

**Figure 2:**
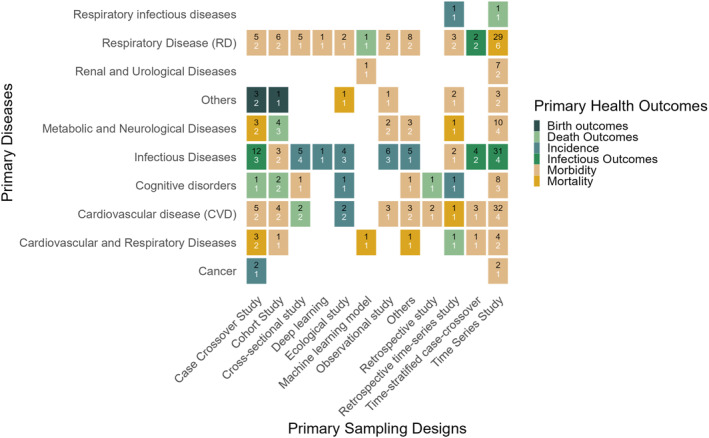
Tile plot of the distribution of primary health outcome by sampling design across major disease categories in distributed lag nonlinear model (DLNM) research. Each tile displays two values: the total number of studies using a specific sampling design for a given disease (top black number) and the number of studied health outcomes (bottom white number). The tile color represents the most‐studied health outcome in each disease‐design pair.

Figure 2 presents a thorough tile plot account of how health outcomes relate to disease classifications and sampling techniques. The numbers reflecting the research frequency and the tile color corresponding to the most‐used health outcomes for each disease‐design pair present the methodological preferences across the literature on the DLNM. This visualization highlights the methodological trends in DLNM applications and emphasizes the health outcomes that are most frequently investigated for each disease type and sampling design.

**Figure 3:**
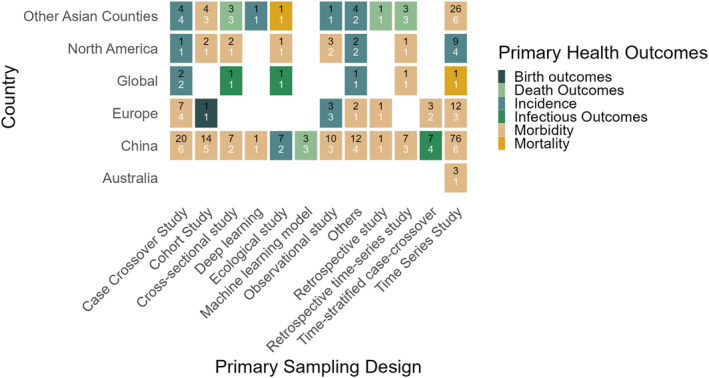
Tile plot of the primary health outcome distribution by sampling design across countries in the distributed lag nonlinear model (DLNM) research. Each tile displays two values: the number of studies using a specific sampling design in that country (top black number) and the number of studies reporting the most frequently used health outcomes (bottom white number). Tile color indicates the most common health outcome for each country‐design pair.

Among time‐series studies (Figure S2 and Table S1 in Supporting Information S1), CVDs appeared in 32 out of 128 time‐series studies (25%), and infectious diseases appeared in 31 of them (24%; black numbers in Figure 3). The studies were associated with four major primary health outcomes: infection outcomes, incidence, morbidity, and mortality (white numbers in Figure 3; see Table S1 in Supporting Information S1 for the outcome details). The most frequent outcomes were infection outcomes in infectious diseases, which were studied in 16 out of 31 (52%) studies, and morbidity in CVDs, which was studied in 27 out of 32 (84%) studies (see Figure 3 with tile colors and Table S1 in Supporting Information S1 for numbers). In the case of RDs, a time‐series design was employed in 29 out of 128 (23%) studies, with a strong preference for mortality outcomes reported in 11 out of 29 (38%) studies. The second most popular sampling technique (n=34) was the case‐crossover design, focusing on three major outcomes: incidence, infection‐related outcomes, and morbidity. The case‐crossover design was most applied in research on infectious diseases (n=12). Generally, morbidity was the most frequently examined health outcome across all diseases when using a time‐series design (61 out of 128 studies; 48%). Notably, RDs and CVDs consistently employed morbidity outcomes across multiple study designs. In contrast, infectious disease research tended toward analyzing incidence outcomes, regardless of the sampling design (Table S1 in Supporting Information S1).

Other sampling designs were less frequently employed but displayed specific patterns. For example, cohort studies (n=21) were primarily applied to chronic diseases, particularly CVDs and RDs, focusing on morbidity. Cross‐sectional studies (n=14) were applied in a variety of disease contexts, often emphasizing prevalence and incidence. Furthermore, observational and retrospective sampling designs were typically employed in studies related to hospital utilization and acute responses. In contrast, ML and deep learning approaches (n=6, combined) represent emerging methods, predominantly employed for predictive modeling of infectious diseases and RDs (see Figure S2 and Table S1 in Supporting Information S1).

### 4.2 Study Plans and Health Outcomes Across Countries

This section presents an analysis of DLNM‐based studies across countries, revealing the consistency and variation in sampling designs and health outcomes (Figure 3). The overwhelming concentration of DLNM research in China, driven by robust data availability, implies that methodological advancements and policy insights derived from DLNM may not be fully transferable or validated in other diverse global contexts lacking similar data infrastructures. The sampling designs, namely the time‐series (128 of 274, 47%) and case‐crossover (34 of 274, 14%) designs, emerged as the most commonly employed methods across regions, with a strong preference for outcomes related to morbidity and incidence, as presented in Table S2 of Supporting Information S1). Although there were regional differences in the study volume across outcomes, morbidity was the dominant health outcome studied in most countries, particularly in large‐scale studies (Figure 3).

The largest concentration of morbidity‐focused research was in China, which contributed the most to the DLNM literature. Therefore, morbidity outcomes, which were reported in 83 (51%) of the 164 Chinese studies across design plans, were the most‐studied outcomes. Another frequently examined health outcome in China was infection‐related outcomes, reported in 22 studies (13%; Table S2 in Supporting Information S1). Other Asian countries, including Japan, South Korea, India, and Vietnam, collectively contributed 49 of the 274 studies, making these countries the second‐largest contributors to the DLNM literature. Notably, the incidence and morbidity outcomes were the most investigated health outcomes in these countries (Table S2 in Supporting Information S1). Although European countries accounted for 28 studies, ranking third in research output and demonstrating a more balanced distribution across morbidity, mortality, and incidence outcomes, morbidity remained the most reported outcome in several study designs (Table 4). In North American studies, a similar emphasis on morbidity was observed, although it was applied to a smaller number of studies (23 of 274) across various sampling designs and outcomes. While the country's analysis varied in the volume of DLNM studies, time‐series designs were the most widely adopted across countries. For example, in China, there were 76 out of 128 time‐series studies with 6 health outcomes, and in Europe, there were 12 out of 128 time‐series studies with 3 health outcomes, particularly for modeling the delayed effects of environmental exposures on public health. These findings suggest a global consensus on the utility of the DLNM for morbidity‐related outcomes, highlighting regional research priorities and methodological preferences (Figure 3, white number on tiles indicate outcome number).

In addition, case‐crossover and cohort studies were most applied in China (20 out of 164 and 14 out of 164, respectively) and other Asian countries (4 out of 49), primarily for long‐term outcomes, such as morbidity and incidence. Cross‐sectional and ecological designs were reported in China (7 out of 164) and North America (2 out of 23), focusing on prevalence and community‐level exposures. Observational and retrospective designs were predominantly found in China and were often applied to hospital‐based data. Emerging approaches (e.g., ML and deep learning) were rarely employed and limited to a few studies in China and Europe, reflecting an increasing interest in advanced modeling in DLNM frameworks (Table S2 in Supporting Information S1).

### 4.3 Exposure Metrics in DLNM Applications

This section of the comprehensive review provides a detailed assessment of the application of the DLNM to study the influence of environmental and atmospheric exposures that have been measured and modeled to assess public health outcomes (Table 2). This analysis describes a broad range of temperature‐related, environmental, and air pollutant variables, offering a complete perspective on the current methodological landscape in DLNM research. There was a noticeable tendency toward temperature‐related measurements, which predominated in the DLNM literature. Among them, the most‐investigated temperature categories were cold (n=37) and hot (n=32), followed by the minimum (n=22), mean (n=28), and maximum temperatures (n=29), respectively. These metrics were typically derived from daily or ambient temperature data, suggesting a preference for standardized, reproducible temperature inputs over inconsistent heatwave definitions.

**Table 2:** Summary of Exposure Measurement Metrics in Distributed Lag Nonlinear Model Studies

Exposure variables	Exposure metrics	No. of studies
Temperature factors		
Ambient temperature	Minimum temperature	22
	Maximum temperature	29
	Cold temperature	37
	Hot temperature	32
Daily temperature	Mean temperature	28
	Minimum temperature	4
	Maximum temperature	4
Weekly temperature	Weekly temperature	4
Hourly temperature	Hourly temperature minimum/maximum	3
Apparent temperature	Apparent temperature	25
Trimester temperature	Trimester temperature	2
Diurnal temperature range	Diurnal temperature range	14
Environmental factors		
Humidity	Low humidity/high humidity	27
Air quality/wind speed	Air quality/wind speed	7
Rainfall	Rainfall	8
Sunshine duration	Sunshine duration	3
Climate change/heat waves	Climate change/heat waves	6
Heavy metals	Heavy metals	1
Meteorological factors	Meteorological factors	24
Wastewater viral signals	Wastewater viral signals	1
Air pollutant factors		
Air pollutants	Gaseous pollutants (NO_2_, CO)	4
	Gaseous pollutants (NO_2_, SO_2_, CO, O_3_)	27
	Particulate matter (PM2.5)	38
	Particulate matter (PM10)	10
	Ozone (O_3_)	14
	Particulate matter (PM10, PM2.5)	50
	Gaseous pollutants (NO_2_, SO_2_)	18
	Carbon monoxide (CO)	4
	Gaseous pollutants (NO_2_)	4
	Gaseous pollutants (SO_2_, NO_2_, CO)	10

*Note.* Some studies examined exposure effects using multiple factors.

Additional metrics, including the apparent temperature (n=25), diurnal temperature range (n=14), and weekly or trimester‐specific temperatures, were employed less often, indicating potential areas for expanded exploration. Although many studies have aimed to define “hot” or “cold” thresholds, the criteria varied considerably. Percentile thresholds (e.g., the 75th to 98th percentiles) were commonly applied to define heat, whereas 0th to 10th percentiles were used for cold, indicating a lack of consensus across the literature (Table 2, temperature factors). Among non‐temperature environmental exposures, humidity was the most frequently modeled, appearing in 27 studies, followed by meteorological factors (n=24), such as wind speed and barometric pressure. Moreover, studies also explored rainfall (n=8), air quality or wind speed (n=7), and climate change indicators (n=6). The assessed studies rarely investigated several factors, such as sunshine duration (n=3), heavy metals (n=1), and wastewater viral signals (*n* = 1), despite their increasing relevance to global environmental health discourse (Table 2, environmental factors).

Further, air pollutants represent a significant focus of exposure assessment in the reviewed DLNM studies. Among these pollutants, particulate matter (PM_2.5_) was examined most, appearing in 38 studies, followed by PM_10_ in 10 studies, and PM_10_ and PM_2.5_ combined in 50 studies.

This emphasis highlights the well‐documented role of fine and coarse particulate matter in contributing to adverse respiratory and cardiovascular health outcomes. In addition to particulates, a broad range of gaseous pollutants have also been evaluated, including gaseous mixtures of NO_2_, sulfur dioxide (SO_2_), and carbon monoxide (CO; n=10), and combinations of NO_2_ and SO_2_ (*n* = 18). Moreover, O_3_ was modeled in 14 studies, and CO and NO_2_ were each individually assessed in four studies (Table 2, air pollutant factors). The variability in how gaseous pollutants were combined or isolated across studies reflects the increasing recognition of the multipollutant exposure paradigm, emphasizing the importance of examining synergistic effects in environmental epidemiology.

Moreover, 10% of the reviewed studies focused on investigating different outcomes in warmer months, whereas 12% of the studies focused on investigating various outcomes according to various diseases in cold months. Approximately 39% of the studies focused on examining exposure effects throughout the year, and 10% of the studies focused on the effects of exposure with various outcomes in the warm and cold seasons (Table 3).

**Table 3:** Number and Percentage of Articles Evaluated by Season

Season	Count (%)
Studies conducted only in summer/warm/hot months	29 (10%)
Studies conducted in winter/cold months	34 (12%)
Studies conducted in warm and cold months	30 (10%)
Studies conducted in duster/flu/flood seasons	8 (3%)
Studies conducted in spring/hot/cold months	15 (5%)
Studies conducted in dry/wet/rainy seasons	7 (3%)
Studies conducted in all four seasons	112 (39%)
Season not specified	52 (18%)
Trimester	2 (0.7%)

### 4.4 Disease Subtypes Assessed in DLNM Studies

The analysis of the focus on primary diseases revealed that CVDs, infectious diseases, and RDs were studied most frequently, each accounting for 26% of the sample (Table 4). This distribution reflects the strong epidemiological relevance of these diseases in the context of environmental exposures, particularly temperature and air pollution, which were the dominant variables in the reviewed literature (Table 6). In contrast, cognitive disorders (5%), metabolic and neurological diseases (8%), and renal and urological diseases (3%) appeared less frequently, indicating underexplored areas for DLNM application. Similarly, cancer (2.9%) and other diseases (4%) were underrepresented, despite well‐established environmental risk factors (e.g., long‐term pollutant exposure and extreme climatic conditions). These gaps highlight opportunities for expanding DLNM research to assess delayed and non‐linear exposure effects across a broader range of disease outcomes.

**Table 4:** Summary of Primary Diseases Assessed in Distributed Lag Nonlinear Model Studies

Primary diseases	*Disease Subtypes*	*n* (%) (*N =* 274)
Cancer	Breast(1), Colon(1), Gastric(1), Lung(2), Thyroid Carcinoma (3)	8 (2.9%)
Cardiovascular diseases	Cardiac(1), Acute Aortic(2), AMI(2), Angina(1), Arrhythmias(2), Hypertension(1), CVDs(36), Stroke(4), Dyslipidemia(1), General Mortality(5), ILD(1), Respiratory and CVD(15)	71 (26%)
Cognitive disorders	Depression(1), Mental Disorders(5), Mental Health(4), Suicide (4)	14 (5.1%)
Infectious diseases	Brucellosis(1), COVID‐19(14), COVID‐20(1), Dengue(3), Diarrhea(2), HFMD(2), Infection(20), Influenza(8), Kawasaki(1), Malaria(2), Myocardial (1), Pneumonia(3), Scarlet Fever(3), Tuberculosis(10)	71 (26%)
Metabolic and neurological diseases	Alzheimer's(1), Dementia(1), Epilepsy(1), Gestational Diabetes(6), Ischemic Stroke(5), Seizures(1), Trauma related Injuries(3)	18 (6.6%)
Other diseases	Allergic(3), Preterm Birth(2), Reproductive Health(1), Various(4), Various Illenesses(1), Viral diseases(2)	13 (4.7%)
Renal and urological diseases	Renal Diseases(6), Urolithiasis(2)	8 (3%)
Respiratory diseases	Respiratory(46), Medical Emergencies(3), Lung Function(1), COPD(2), Childhood Asthma(3), Bronchiectasis(1), Asthma(15)	71 (26%)

To provide greater granularity, Table 4 presents these primary disease categories as subtypes. For instance, the CVD group includes stroke (n=4), hypertension (n=1), acute myocardial infarction (n=2), and cardiac arrest (n=1), with several studies addressing overlapping conditions, including CVD with respiratory outcomes (n=15). The RD category features commonly studied conditions, such as asthma (n=15), COPD (n=3), and childhood asthma (n=3). Moreover, infectious disease studies cover a wide range of pathogens and conditions, including COVID‐19 (n=14), influenza (n=8), dengue fever, tuberculosis, and HFMD, all of which have demonstrated sensitivity to climate variables, such as temperature and humidity (Table 4). Additional subcategories highlight various conditions, such as gestational diabetes, ischemic stroke, mental disorders, urolithiasis, and preterm birth, indicating the versatility of the DLNM in modeling delayed health effects across diverse systems and populations. However, the relatively limited number of studies in these areas suggests the need for more targeted research to fully apply the potential of the DLNM in diverse health domains.

## 5 Discussion

To our knowledge, this review presents the first comprehensive examination of the relationship between DLNM studies and public health outcomes across diverse geographical settings. Previous systematic and scoping reviews have predominantly concentrated on specific health outcomes related to particular exposures, such as the effects of raised ambient temperatures on maternal, fetal, and neonatal outcomes (Dalugoda et al., 2022). Other reviews have assessed heat impacts across various geographical contexts and health outcomes (Graffy et al., 2024). Another study focused on aspects of the study design that critically influence the interpretation of the research findings (Syed et al., 2022). Additional evaluations have identified methodological approaches common in heat impact studies with pregnancy outcomes (Lakhani et al., 2024; Mehta et al., 2023). Moreover, a scoping review highlighted mixing approaches to investigate the health effects of persistent organic pollution exposures (Pan et al., 2024). Some systematic reviews published statistical values in meta‐analyses (Gao et al., 2019; Xu et al., 2019). However, no previous research has synthesized the broader scope of the DLNM literature concerning public health outcomes, including a detailed examination of sampling strategies, diseases, and specific outcomes.

The analysis indicated that the DLNM is the most‐employed analytical framework, regardless of the studied health outcomes. Studies have applied this model in combination with various exposure variables, including temperature, environmental factors, and air pollutants, in several sampling study designs. Furthermore, this review emphasizes the significance of study design characteristics, which are critical for understanding outcomes and improving study comparability in prospective meta‐analyses or systematic reviews. This evaluation discovered that case‐crossover, time‐series, and cohort study designs were most popular among several statistical models, including the DLNM with Poisson distribution regression, general linear models, and logistic regression models.

Furthermore, the review clearly demonstrates a significant geographical imbalance in the application of the DLNM, which directly influences our understanding of model performance in various contexts. As detailed in the findings and summarized in Table 6, this research has overwhelmingly been concentrated in China (164 studies), followed distantly by Europe (28) and North America (23). This success is primarily driven by the availability of robust, long‐term environmental and health registry data in these regions. Regarding how the models perform across these spatial contexts, the critical finding is not that the DLNM technique is less valid elsewhere but that the estimated exposure–response relationships are highly specific to the context.

### 5.1 Fundamental Assumptions

This review employs a systematic approach to examine the application of the DLNM to study environmental exposure and public health outcomes. Moreover, 274 studies published from January 2020 to November 2024 were analyzed, spanning a diverse range of geographical settings across 36 countries, predominantly in China, Europe, and North America. The studies revealed significant variability in exposure metrics, including temperature extremes, environmental pollutants, and diverse exposure windows, which are crucial for accurate public health assessments. A range of epidemiological study designs, such as time‐series, case‐control, ecological studies, prospective, and cross‐sectional designs, were employed to evaluate the effects of environmental exposures.

1. *Public Health Outcomes*: The most frequently studied outcomes included hospital admissions, morbidity, and mortality associated with RDs, CVDs, and infectious diseases. These outcome measures reflect the dominant burden of environmentally sensitive health conditions in the reviewed settings.
2. *Epidemiological Models*: The focus on the DLNM highlights its utility in capturing complex temporal trends and relationships between environmental exposure and health metrics, improving the understanding of the lagged effects of such exposures.
3. *Comparative Study Design Features*: The diversity of sampling and study designs highlighted in this review reinforces the importance of the methodological context when interpreting DLNM‐based findings. The comparative analysis of the study design features across geographic regions and health outcomes provides a valuable foundation for future meta‐analyses and cross‐study synthesis.

According to the findings, the most significant health outcomes (associated with CVDs, RDs, and infectious diseases), which are common occurrences in the main study settings, are switched around in different exposure effect studies. The influence of environmental exposures is highlighted in the small number of research studies that assessed health consequences, including mental health disorders, neurological conditions, and ecological problems.

### 5.2 Modeling and Interpreting the Temporal Variability of Effects

This synthesis reveals that the health effects of environmental exposures are not static but vary significantly with the temporal context, a concept known as temporal effect modification. This concept is a central theme in the reviewed DLNM literature. Table 3 underscores this finding, revealing that over 60% of the studies explicitly considered the seasonality by analyzing warm or cold months or the entire year. This practice is driven by evidence that the magnitude of an effect can changes dramatically depending on the time of year. For instance, studies we reviewed (Tan et al., 2023) on seasonal HFMD risk and (Kim et al., 2024) on the dangers of unseasonal heat, use the DLNM to demonstrate that the timing of exposure is as critical as its intensity. Researchers have captured this variability primarily via seasonal stratification (fitting separate models for warm and cold seasons) (Kim et al., 2021) or by incorporating exposure metrics that represent the daily volatility (e.g., the diurnal temperature range), which was employed in 14 studies (Table 2). The interpretation of this variability is multifaceted, often attributed to diverse factors, such as physiological acclimatization, behavioral adaptations (e.g., air conditioning use), and the presence of seasonal coexposures (e.g., influenza). This finding demonstrates that the DLNM framework is invaluable for estimating the main effect of an exposure and characterizing how that risk dynamically evolves, providing crucial evidence for targeted public health interventions, such as seasonal heat‐health action plans.

### 5.3 Cross‐Classified DLNM Applications by Diseases Categories

To synthesize outlines in DLNM applications across the 274 reviewed studies, we classified research outputs according to five major dimensions: disease category, exposure metric (temperature, air pollutants, and other environmental factors), study design (e.g., time series, case‐crossover, and cohort), effect size, and geographical region. This cross‐tabulation reveals dominant trends and underexplored combinations of environmental risk and health outcomes (see Table 5).

**Table 5:** Comparison of Distributed Lag Nonlinear Model Studies by Disease, Exposure Metric, Effect Size, Study Design, and Country

Diseases category	Exposure metrics			Most‐used sampling design				Countries					Effect sizes				
	Temperature	Air pollutants	Other factors	Time series	Case‐crossover	Cohort study	Other designs	China	Europe	North America	Other Asian countries	Other countries	RR	OR	Rate R	HR	Others
CVD	39	27	5	38	8	5	20	46	8	4	9	1	30	19	13	6	3
RD	23	31	17	30	5	7	29	45	7	7	1	0	27	26	7	3	8
Infectious Disease	30	17	24	30	12	2	27	39	9	6	13	4	31	16	5	8	11
Metabolic and Neurological	10	7	7	10	3	4	7	14	3	1	4	2	11	3	1	2	1
Cognitive Disorders	7	3	3	6	2	1	4	7	0	2	4	0	5	3	3	3	0
Renal and Urological	6	1	1	7	0	0	1	7	1	0	0	0	5	2	0	0	1
Other Diseases	4	5	2	3	3	1	3	5	1	2	1	1	8	3	0	0	2
Cancer	0	7	1	2	2	2	2	7	0	1	0	0	1	6	0	0	1

*Note.* RR, relative risk; OR, odds ratio; HR, hazard ratio; CVD, cardiovascular disease; RD, respiratory disease.

#### 5.3.1 Cardiovascular Diseases

These diseases were the most consistently studied using the DLNM, especially in China (n=46), where researchers extensively explored temperature (n=39) and air pollutant exposures (n=27). The predominant approach was the time‐series design (*n* = 38) using the relative risk (n=30) or odds ratio (OR; n=19) as effect measures, reflecting an emphasis on capturing long‐term exposure effects. Fewer studies applied case‐crossover (n=8) or cohort designs (n=5) or other effect sizes, such as the hazard ratio (n=6). Additional research is necessary for other environmental variables, such as humidity and wind (n=5), which remain underused (Table 5 and Figure S1 and Table S8 in Supporting Information S1). Table 6 reveals that studies have focused on temperature (n=39) and air pollutants (n=27), using time‐series designs to capture short‐term risks. The non‐linear function in the DLNM is critical for identifying specific temperature or pollution thresholds that trigger events, such as myocardial infarction or stroke. In contrast, the lag function can determine whether the risk is immediate (a lag of 0–1 day) or slightly delayed. For instance, a study (An et al., 2025) which used the DLNM to link ambient temperatures to emergency ambulance dispatches for CVD in Chongqing, exemplifying how this model provides actionable evidence on the immediate cardiovascular burden of extreme weather.

**Table 6:** Reported Strengths and Limitations of the Distributed Lag Nonlinear Model

Category	Strengths	Limitations
Temporal modeling	Effectively captures non‐linear and lagged associations between environmental exposures and health outcomes (Gasparrinia et al., 2010)	Highly sensitive to lag window specifications and degrees of freedom; potential for overfitting (Schwartz, 2000)
Interpretability	Provides intuitive graphical outputs (e.g., lag–response surfaces) that enhance the understanding of complex associations (Gasparrini, 2014)	Interpretation becomes challenging with interaction effects or multidimensional lag (Wilson et al., 2017)
Versatility	Applicable across a wide range of exposures (temperature and pollutants) and outcomes (morbidity and mortality) (Gestro et al., 2017)	Limited use in high‐dimensional or multiexposure models; lacks standardization in complex contexts (Michetti et al., 2022)
Study design integration	Compatible with time‐series, case‐crossover, and cohort designs (Entezari & Mayvaneh, 2019)	Difficulties in adjusting for time‐varying confounders or policy shifts in real‐world applications (Achebak et al., 2023)
Computational efficiency	Easily implemented in software (e.g., the *R* DLNM package); (Gasparrini, 2011) suitable for large environmental data sets (Gao et al., 2023)	Advanced extensions (e.g., the Bayesian DLNM or hybrid ML‐DLNM models) remain computationally intensive and underused (Quijal‐Zamorano et al., 2024)
Modeling flexibility	Supports user‐defined splines for exposure–response and lag–response functions (Nguyen et al., 2024)	Requires careful specification of degrees of freedom to avoid model misspecification (Gasparrini, 2011)
Risk estimation	Accurately models short‐ and long‐term cumulative effects, outperforming simpler lag methods (Luo et al., 2022)	Estimates may vary depending on the exposure metrics and lag structure (Neophytou et al., 2018)
Communication of results	Three‐dimensional visualizations support clearer public health messaging and risk communication (Gasparrinia et al., 2010)	Lack of consensus on how to standardize visualization and interpretability across contexts

*Note.* DLNM, distributed lag nonlinear model.

#### 5.3.2 Respiratory Diseases

Research on the DLNM related to respiratory diseases has predominantly been conducted in China (n=45). Among these studies, air pollutants (*n* = 31) were more frequently examined than temperature (n=23), likely due to their significant correlation with respiratory morbidity. Various research designs were employed, including time‐series (n=30), cohort (n=7), and case‐crossover (n=5) methods, using the relative risk (n=27) and OR (n=26) as effect measures. The predominant exposure of interest was air pollutants (n=31) over temperature (n=23), reflecting the direct influence of pollutants on the respiratory system. The DLNM is indispensable in this field for clarifying the complex temporal dynamics of respiratory exacerbations. For example, the model can reveal whether an asthma attack is triggered by the same‐day ozone exposure or the cumulative effect of particulate matter over several days (see Table 5 and Figure S2 and Table S8 in Supporting Information S1). A study (Ma et al., 2018) examined the effect of air pollution on emergency room admissions for RDs, revealing the ability of the model to provide clear evidence on the lagged health effects of poor air quality, informing public health alerts and clinical advice.

#### 5.3.3 Infectious Diseases

Infectious disease modeling with the DLNM has been distributed globally, with studies from China (n=39), other Asian countries (n=13), Europe (n=9), and North America (n=6). Exposure variables were more diverse, including temperature (n=30), air pollutants (n=17), and other environmental factors (n=24), such as rainfall and humidity. The time‐series design (n=30) was dominant, though case‐crossover (n=12) approaches have increasingly been used to model outbreaks. Infectious disease models commonly rely on short‐term lag structures, captured via various sampling designs and effect sizes, such as the relative risk (n=31), OR (n=16), and hazard ratio (n=8). Only two studies employed cohort designs, indicating an opportunity for future longitudinal infectious disease modeling using the DLNM (see Table 5 and Figure S3 and Table S8 in Supporting Information S1).

**Figure 4:**
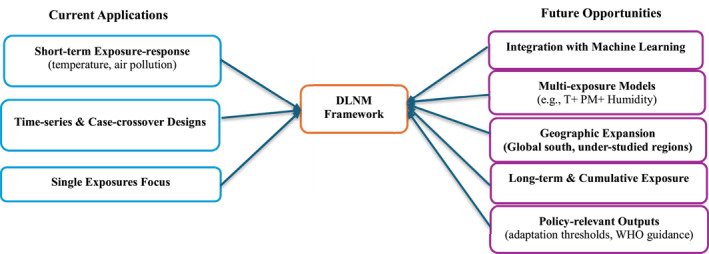
Conceptual overview of DLNM applications.

To better understand the utility of the DLNM in infectious disease epidemiology, we classified the diseases studied in the review by their primary transmission routes. The analysis of the 71 infectious disease studies (Table 4) reveals that the DLNM has been most frequently and effectively applied to model airborne and vector‐borne diseases. The airborne category is prominent, with numerous studies on COVID‐19 (n=14) and influenza (n=8; Figure 4). For these pathogens, the DLNM is exceptionally well‐suited to capture the complex, non‐linear, and delayed effects of meteorological factors, such as temperature and humidity, on virus viability and human social behavior. Similarly, vector‐borne diseases represent another area of high influence, with studies on dengue fever being a critical example. The strength of the DLNM lies in its capacity to model the crucial biological lag between environmental conditions (e.g., rainfall and temperature) and disease incidence, driven by the life cycle of the vector, a dynamic captured well (Lowe et al., 2018). Notably, although theoretically applicable, classic waterborne diseases were less prominent in the review, suggesting that the most successful applications of the DLNM have been for diseases where environmental drivers have complex, non‐linear, and significantly lagged effects on the pathogen or its vector, making this modeling approach uniquely robust.

#### 5.3.4 Metabolic and Neurological Disorders

This group included 14 studies from China, and smaller contributions from Europe (n=3), North America (n=1), and other Asian countries (n=4). The time‐series approach (n=10) again dominated, although case‐crossover (n=3), cohort (*n* = 4), and other designs (*n* = 7) were more evenly distributed compared to other disease categories. The primary exposure investigated was temperature (n=10), with studies exploring its effect on various conditions, such as diabetes and mental health disorders. The strength of the DLNM in this domain is its ability to assess hypotheses regarding less‐direct biological pathways. For example, a heatwave might not cause a metabolic or neurological event directly, but it could disrupt physiological regulation over several days. In a case‐crossover study (Zhang et al., 2020), which used the DLNM to evaluate the prolonged and delayed effects of low temperatures on various mental disorders, demonstrating the capacity of the model to uncover complex temporal patterns in these less‐explored but critical health outcomes. This finding suggests increasing methodological diversification in this domain (see Table 5, Figure S4 and Table S8 in Supporting Information S1).

#### 5.3.5 Cognitive Disorders

Seven studies have been published from China and four studies from other Asian countries, with a few contributions from North America (n=2). Most research has focused on temperature exposure (n=7) and employed time‐series (n=6) or other designs (n=4), with limited adoption of case‐crossover and cohort frameworks. This research is crucial for understanding the links between environmental stressors and acute mental health crises. The DLNM is the ideal tool for this work because it can identify the specific lag period during which a heat event might trigger a psychiatric emergency. The Korean study (Lee et al., 2018) provided a clear example using the DLNM to pinpoint that the strongest association between hot temperatures and emergency admissions for mental diseases occurred within a 0–4 days lag window (see Table 5; Figure S5 and Table S8 in Supporting Information S1).

#### 5.3.6 Renal and Urological Diseases

This category was among the least‐studied in the review, with only eight articles, the vast majority of which were from China (n=7) and focused on temperature exposure (n=6). This geographical and topical concentration suggests a specific research interest in the effects of heat‐related dehydration on kidney function. The biological link is direct, making the DLNM an excellent tool for quantifying the risk of various conditions, such as acute kidney injury or urolithiasis (kidney stones) following heat events. The lag dimension of the model can distinguish between immediate effects from acute dehydration and cumulative effects that build over a multiday heatwave. Although we found a systematic review (Liu et al., 2021) that confirmed the importance of underscoring the need for more DLNM‐based research to provide precise risk estimates for this highly vulnerable organ system (see Table 5; Figure S5 and Table S8 in Supporting Information S1).

#### 5.3.7 Other Diseases and Cancers

This heterogeneous group included various conditions, such as gout, Kawasaki disease, and preterm birth. Contributions came from China (n=5) and scattered efforts across Europe, North America, and Asia. The study designs were mixed, with three time‐series, case‐crossover, and alternative designs, reflecting experimental approaches in this underdefined category. The studies investigated temperature (n=4) and air pollutants (n=5), although rarely in combination (see Table 6; Figure S5 and Table S8 in Supporting Information S1). In addition, the study of cancer predominantly employed the DLNM, particularly concerning air pollutant factors, with most research (n=7) conducted in China.

Notably, DLNMs have underpinned several influential multi‐country studies that shaped climate and environmental health policy. For instance, Gasparrini et al. (2015) demonstrated that moderate cold accounted for more deaths worldwide than extreme heat, reframing climate‐related health risk assessments. Similarly, Yang et al. (2024) identified temperature variability as an independent risk factor for mortality across diverse settings. DLNMs have also been central to large‐scale air pollution studies (Yu et al., 2024), providing robust evidence that informed WHO air quality guidelines. These examples illustrate how the flexibility of DLNM in capturing delayed and nonlinear associations has directly advanced public health understanding and policy.

### 5.4 Significance of Lag Structures by Disease Category and Public Health Implication

Importantly, the critical strength of the DLNM framework, highlighted throughout this review, is its ability to identify disease‐specific lag structures with distinct public health implications. For acute outcomes, such as CVDs, RDs, and mental health events, the critical lag structures are typically immediate, spanning just a few days (e.g., lags 0–4). This finding is exemplified by studies on CVD‐related ambulance calls (An et al., 2025) and emergency admissions for mental disorders (Lee et al., 2018), where exposures function as direct triggers. The implication for public health is that rapid response systems, such as real‐time heat warnings and air quality alerts, are needed. In contrast, outcomes involving complex biological processes, such as vector‐borne infectious diseases and maternal/neonatal health, exhibit much longer lags of weeks or even months. For example, the lag in the incidence of dengue fever reflects the life cycle of the vector post‐rainfall (Lowe et al., 2018), whereas in maternal health, lags correspond to specific “critical windows” of fetal development during gestation, such as the 27th to 33rd gestational weeks for PM_2_._5_ exposure (Wu et al., 2018). These extended lags are invaluable because they allow for proactive and predictive interventions, such as timely vector control or targeted advice for pregnant women. The lag dimension identified by the DLNM is a statistical parameter and a critical piece of evidence that directly informs whether public health strategies should be reactive or prospectively planned.

### 5.5 Gap and Future Recommendations

While DLNM has demonstrated robust capabilities in modeling acute and short‐to‐medium term health outcomes, particularly morbidity, its application for assessing long‐term or cumulative exposures remains significantly underutilized (Figure 4). This represents a critical gap, as many environmental health effects, such as those related to chronic disease development, unfold over extended periods and require more sophisticated longitudinal applications. A critical methodological gap remains in the integration of the DLNM with other advanced statistical methods (Figure 4).

The literature highlighted the findings of environmental exposure effects using the DLNM with traditional statistical frameworks, such as Poisson regression, generalized additive models, linear regression, and logistic regression models (Graffy et al., 2024). However, relatively few studies have explored collaborations with ML, deep learning, or Bayesian inference approaches (Guo, Liu, et al., 2021; Hwang et al., 2023; Liu et al., 2024; Sun et al., 2023). Although the DLNM model has robust capabilities in analyzing temporal relationships, the exploration of its potential combination with ML algorithms, Bayesian approaches, and neural networks remains limited. This gap in methodological integration represents a missed opportunity to enhance the predictive capabilities of the model and offer more detailed insight into exposure–health relationships (Mork & Wilson, 2022).

The findings are inconsistent due to this gap; hence, we recommend more research that explores combining the DLNM with advanced statistical models to provide promising results in capturing complex time‐based patterns. The implementation of deep neural networks and ML methods can enhance the analytical capabilities of traditional statistical methods. This integration has achieved remarkable success in detecting and analyzing complex patterns, with studies finding significantly enhanced predictive performance compared with conventional approaches (Lu et al., 2020; Qureshi et al., 2025; Thomas, 2024; Zhong et al., 2023). The emergence of the spatial Bayesian DLNM marks another significant development in the field. These models offer a sophisticated framework for estimating reliable small‐area lagged non‐linear associations (Quijal‐Zamorano et al., 2024). The Bayesian framework allows for more robust uncertainty quantification while incorporating spatial dependencies that traditional DLNM might overlook.

The development of distributed‐lag models using Bayesian kernel machine regression is an innovative approach to handling complex exposure–response relationships (Wilson et al., 2022). This method accounts for non‐linear associations and temporal dependencies simultaneously, offering new possibilities for analyzing environmental health data. The integration of kernel methods with the DLNM offers greater flexibility in modeling complex interactions while ensuring interpretable results. Such integrations of the DLNM with advanced statistical methods represent a significant step in environmental health research. These developments enhance the ability to analyze complex exposure–response relationships, addressing their computational, methodological, and standardization challenges, which are crucial for advancing their application, providing more robust and reliable results. With the advancement of computational capabilities, we can expect further innovations in these integrated approaches, yielding more sophisticated and practical applications in environmental health research.

### 5.6 Limitations of the Comprehensive Review

This review has some limitations due to its comprehensiveness. Although this evaluation followed PRISMA guidelines, some papers might have been unintentionally eliminated due to search terms or the review procedure. To the best of our knowledge, no previously published search phrase has been developed for this review type, which could be ascribed to a lack of consensus or defined process for the query. However, this review is the most wide‐ranging of its kind; thus, the included studies are likely to reflect the scope of the relevant English literature accurately, despite the authors' lack of translation assistance. We did not include hand‐searched publications or gray literature, such as government reports, conference papers, blogs, interviews, dissertations, and theses; therefore, we may have missed specific research that did not surface in the keyword search. The categorical method might have concealed exceptional cases when presenting various study characteristics, such as the design setting, statistical analysis, outcomes, and exposure. The review was not limited to examining the specific effects of elevated temperatures on adverse maternal outcomes, pulmonary tuberculosis, CVDs, RDs, or kidney disease. Instead, this study broadly investigated the effects of various environmental exposures on diverse diseases using the DLNM statistical approach. Additionally, we did not explore potential effect modifiers (e.g., air pollution), which have been examined in some earlier systematic reviews (Cheng et al., 2019; Liu et al., 2021).

### 5.7 Structural and Methodological Advantages of the DLNM Over Traditional Models

The widespread adoption of the DLNM, as documented by the 274 studies in this review, is a direct result of its unique ability to overcome the structural and mathematical limitations in previous time‐series models. This literature review found that early epidemiological approaches (e.g., single‐lag models or moving averages), as critiqued by Samoli et al. (2005), often oversimplify temporal dynamics. These methods either arbitrarily focus on a single‐lag model or assume an implausible constant effect across an entire lag window, failing to capture the true, flexible, and distributed nature of health effects in response to environmental exposures. However, a seemingly intuitive advancement—simultaneously applying flexible non‐linear functions (e.g., splines) to each lag (for example, y∼s(x_t)+s(x_t−1)+...)—is invalidated by a severe mathematical problem: multicollinearity because an environmental exposure on one day is highly correlated with its value on adjacent days, the model cannot distinguish the independent effects of the individual spline terms, leading to statistically unstable, unreliable, and uninterpretable coefficient estimates.

The core innovation of the DLNM framework, which was introduced by (Gasparrinia et al., 2010) that's an elegant solution to this multicollinearity problem by creating a cross‐basis matrix. This unique structure is formed by combining two sets of basic functions: one to capture the non‐linear shape of the exposure–response relationship and another to model the flexible shape of the lag–response relationship. This fundamental design allows the model to estimate a complex, two‐dimensional exposure–lag–response surface using relatively few parameters, ensuring statistical stability while maintaining high flexibility. As highlighted in Table 6 (detailing the strengths of the DLNM), this cross‐basis provides unparalleled advantages. This standard framework is the only one that can simultaneously model the non‐linear exposure‐response and flexible lag–response dimensions without succumbing to multicollinearity. The direct output, a comprehensive 3D surface, offers an intuitive and biologically meaningful visualization of how the risk from a specific exposure level evolves over all relevant lags, yielding insight impossible to achieve with simpler, one‐dimensional models.

The quantitative findings further underscore the unique utility of the DLNM. As depicted in Figure S6 of Supporting Information S1, the DLNM was employed as the sole modeling approach for the exposure‐lag response in 183 studies (66.8%), demonstrating its stand‐alone robustness. In other instances, the DLNM was applied in conjunction with other generalized linear models, such as Poisson (n=33), logistic (n=14), or linear regression (n=13). This pattern reflects a crucial distinction: these other models typically address the statistical distribution of the outcome variable (e.g., suitable for count data in Poisson regression), whereas the DLNM uniquely and specifically handles the complex temporal and non‐linear structure of the environmental exposure. This customary practice of integrating the DLNM with a base generalized linear model highlights its specialized and indispensable contribution to accurately characterizing intricate exposure–health relationships.

## 6 Conclusion

This review offers a thorough overview of the applications of the DLNM in environmental epidemiology, demonstrating the adaptability of this model for simulating the non‐linear and delayed effects of environmental exposures on diverse health outcomes. Although the majority of applications have concentrated on infectious, respiratory, and cardiovascular diseases, this review also finds a significant gap in DLNM studies that address neurological, metabolic, renal, and mental health conditions, which are becoming increasingly important to assess environmental exposures owing to climate change.

Future studies should focus on three critical areas to progress advancements in this discipline. First, given the wide range of definitions and modeling of short‐ and long‐term delays occurring in the existing work, a comprehensive review of lag structure requirements should be conducted. Across exposure‐outcome domains, standardizing lag selections would improve the repeatability and comparability of the DLNM‐based results. Second, especially in multiexposure or high‐dimensional data settings, integrating the DLNM with sophisticated modeling frameworks (e.g., neural networks, Bayesian hierarchical models, and ML algorithms) can enhance the adaptability, interpretability, and predictive performance of exposure–response estimation. For instance, kernel machine‐based lag models and the spatial Bayesian DLNM offer promising instruments to capture complex nonlinear relationships and spatial dependencies. Third, methodological development should focus on expanding the DLNM to capture multiexposure interactions and time‐varying confounders, which remain under‐addressed in the existing literature. Additionally, research should focus on developing standards for best practices for model implementation, such as defining exposure thresholds, setting lag windows, and specifying spline parameters.

Overall, the DLNM has a robust foundation for temporal exposure–response modeling, but its full potential can only be realized via deeper methodological integration, domain expansion, and standardized reporting practices. These advances are critical for producing highly effective, actionable evidence to inform environmental health policy and climate‐resilient public health strategies.

## Conflict of Interest

The authors declare no conflicts of interest relevant to this study.

## Supporting information

Supporting Information S1

## Data Availability

The data supporting this review are derived from publicly available databases, including PubMed, Web of Science, and Scopus. No new data sets were generated for this study. All referenced study's data sets are available and can be downloaded in Shafqat and Park (2025a). The Supporting Information S1, including additional analyses and supporting figures, are provided in Supporting Information S1. The *R* code used for data analysis is available in Shafqat and Park (2025b).
