# Supplementary material for: Broad Applications of Distributed Lag Non‐Linear Model in Public Health: A Comprehensive Review
Source: Geohealth. 2025 Dec 9;9(12):e2025GH001608. doi: 10.1029/2025GH001608 (PMC12686970; doi:10.1029/2025GH001608)
Supplement: Supplementary file 1 — Supporting Information S1 [file GH2-9-e2025GH001608-s001.docx]

*GeoHealth*

Supporting Information for

Broad Applications of Distributed Lag Non-linear Model in Public Health: A Comprehensive Review

Ambreen Shafqat^1^, Eunsik Park^1*^

^1^Department of Mathematics and Statistics, Chonnam National University, Gwangju, South Korea

Email: ambreenshafqat@jnu.ac.kr

*Corresponding author email: espark02@jnu.ac.kr

**Contents of this file**

Text S1 to S4

Figures S1 to S6

Tables S1 to S8

Text S1.

A comprehensive literature search was executed across several databases, including PubMed, Scopus, and Web of Science, to identify studies utilizing Distributed Lag Nonlinear Models (DLNM) in the context of environmental health outcomes. The search strategy incorporated keywords related to temperature, air pollution, humidity, and specific disease outcomes such as mortality, morbidity, and suicide. Duplicates were eliminated, and articles were screened based on their titles and abstracts. Full-text reviews were conducted to ensure methodological relevance. The inclusion criteria comprised: (1) studies employing DLNM or distributed lag methodologies, (2) quantitative analyses of environmental exposure–response relationships, and (3) reported outcomes related to mortality, morbidity, or other health endpoints. Exclusion criteria encompassed reviews, conference abstracts, non-English publications, and studies not employing time-series or case-crossover designs. Data were extracted concerning publication year, study region, exposure type, disease category, and model specifications.

Text S2.

The studies included in this analysis encompassed a broad spectrum of environmental exposures, primarily focusing on temperature, particulate matter (PM2.5 and PM10), humidity, diurnal temperature range (DTR), and air pollutants such as ozone (O₃), nitrogen dioxide (NO₂), and sulfur dioxide (SO₂). The exposure metrics varied among the studies, with the majority utilizing daily mean or maximum temperature and 24-hour average pollutant concentrations. Health outcomes were classified into major disease categories: cardiovascular, respiratory, renal, metabolic, infectious, and mental health outcomes, including suicide mortality. These categories were aligned with ICD-10 classifications. Regional variations in exposure–outcome relationships were examined to account for heterogeneity across different climate zones and population characteristics.

Text S3.

The Distributed Lag Nonlinear Model (DLNM) framework served as the principal methodology for quantifying nonlinear and delayed relationships between environmental exposures and health outcomes. Studies frequently employed quasi-Poisson or Gaussian family regression models within a generalized linear model (GLM) framework. DLNM facilitates the simultaneous modeling of both the exposure–response curve and the lag–response structure through cross-basis functions. Generalized Additive Models (GAMs) and Bayesian hierarchical models were also employed for sensitivity analyses and spatial comparisons. Model selection typically involved minimizing the Akaike Information Criterion (AIC) or utilizing penalized splines to mitigate overfitting. Adjustments for seasonality, long-term trends, and autocorrelation were made using natural cubic splines or time-stratified case-crossover designs.

Text S4.

Regional analyses have demonstrated that the magnitude and configuration of exposure–response associations differ across geographical locations. For instance, tropical and subtropical regions exhibit elevated temperature-related risks, whereas temperate areas display more pronounced associations with cold extremes. Studies conducted in East Asia and Europe frequently report multi-city comparisons, thereby enhancing the generalizability of DLNM findings. The reviewed literature, however, is limited by inconsistent exposure definitions, restricted availability of individual-level covariates, and a publication bias favoring large metropolitan datasets. It is essential to consider these methodological differences when interpreting results. Additionally, the integration of DLNM with machine learning and Bayesian inference represents an emerging trend that may enhance predictive accuracy and reduce uncertainty in future environmental health assessments.


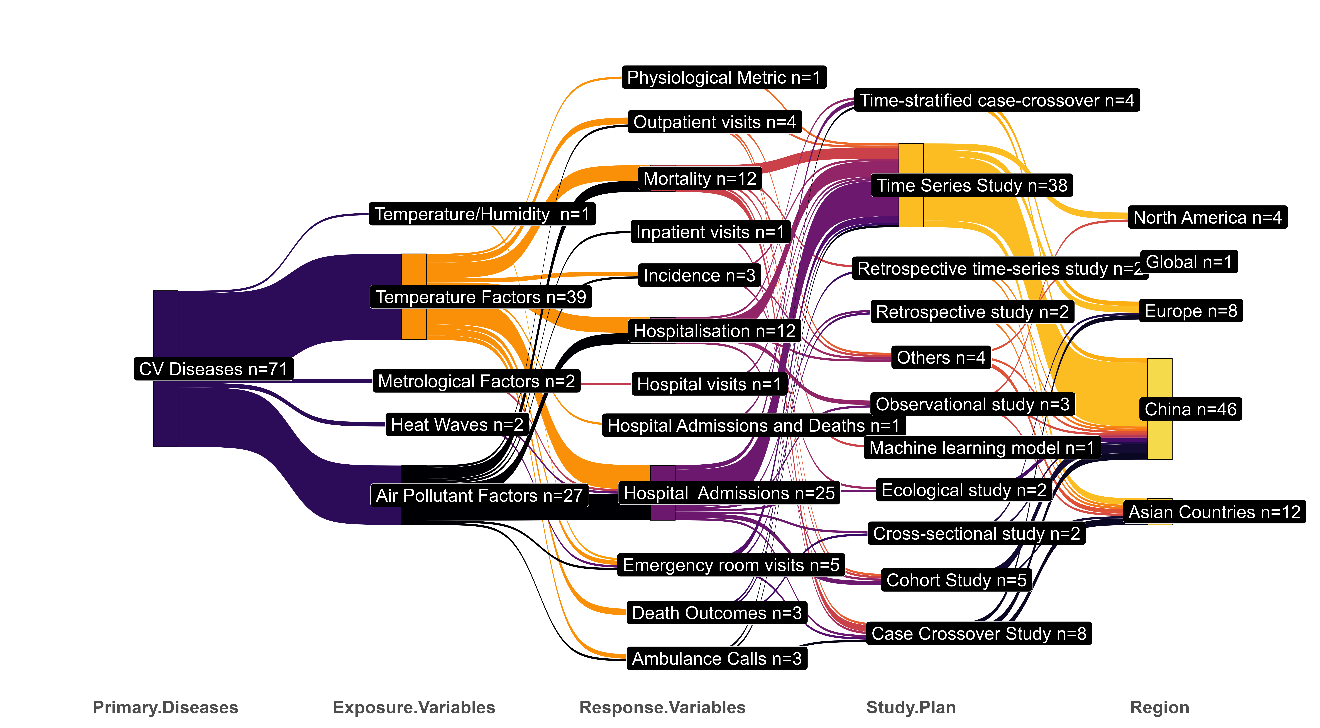


Figure S1. Snaky plot for the CVDs characteristics analysis.


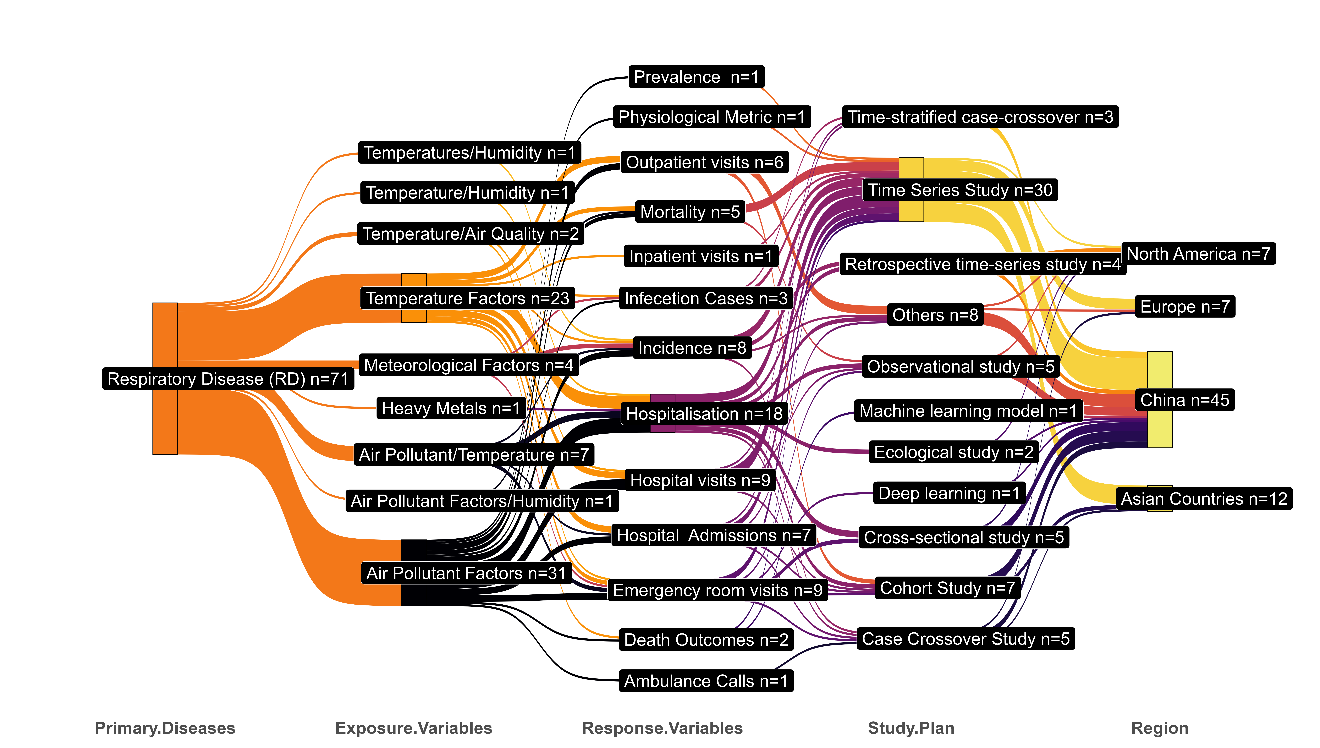


Figure S2. Snaky plot for the RDs characteristics analysis.


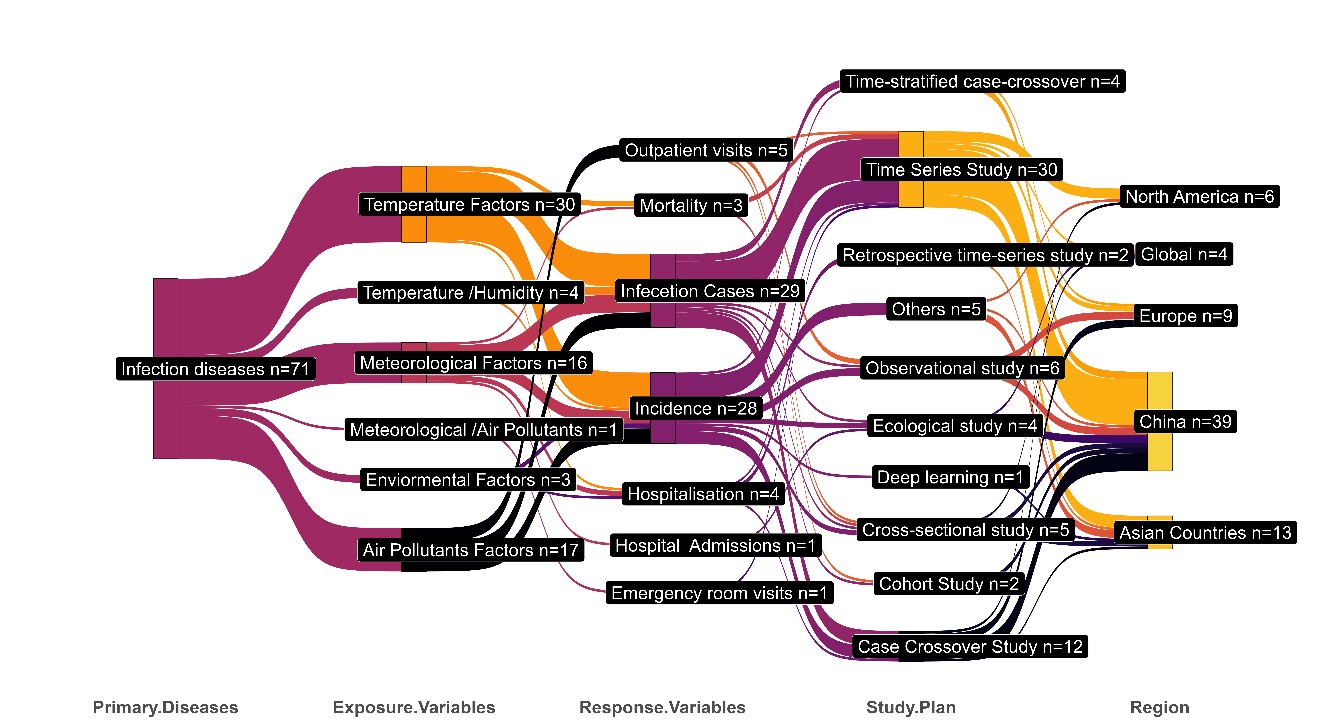


Figure S3. Snaky plot for the IDs characteristics analysis.


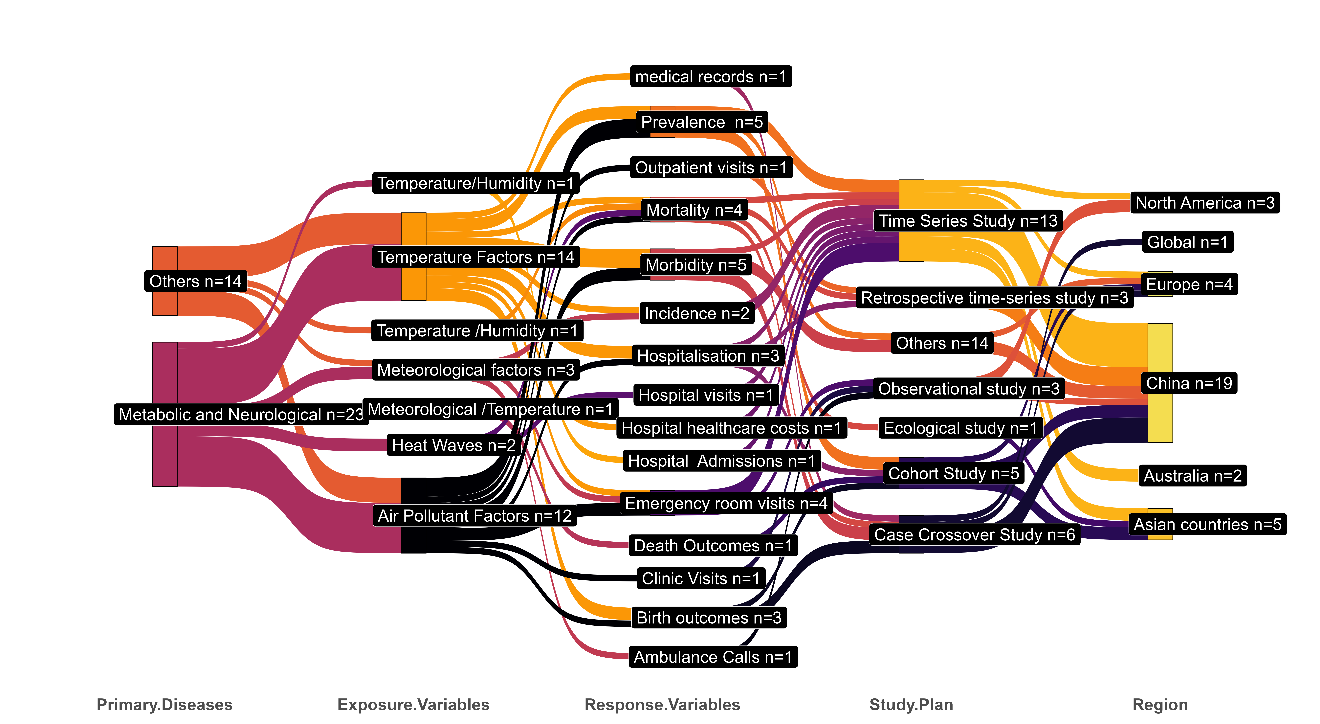


Figure S4. Snaky plot for the Metabolic and Neurological and other diseases characteristics analysis.


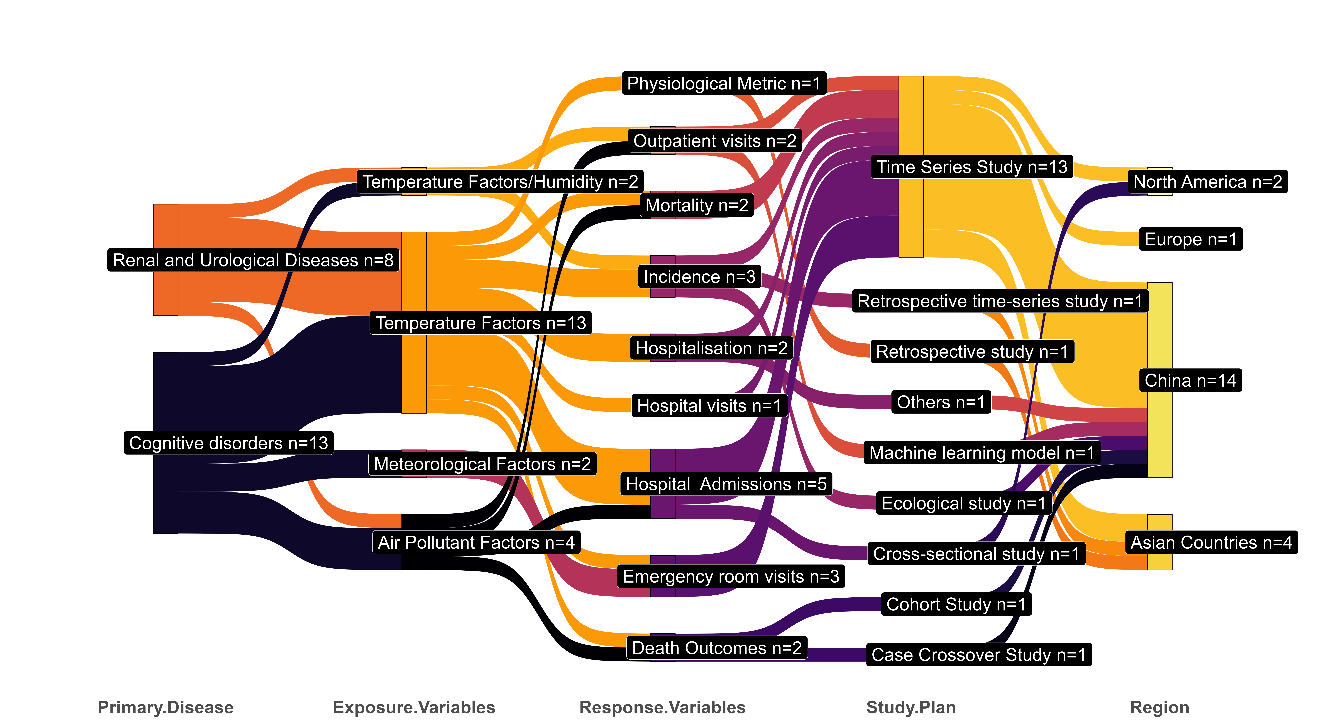


Figure S5. Snaky plot for the Cognitive, Renal, and Urological diseases characteristics analysis.


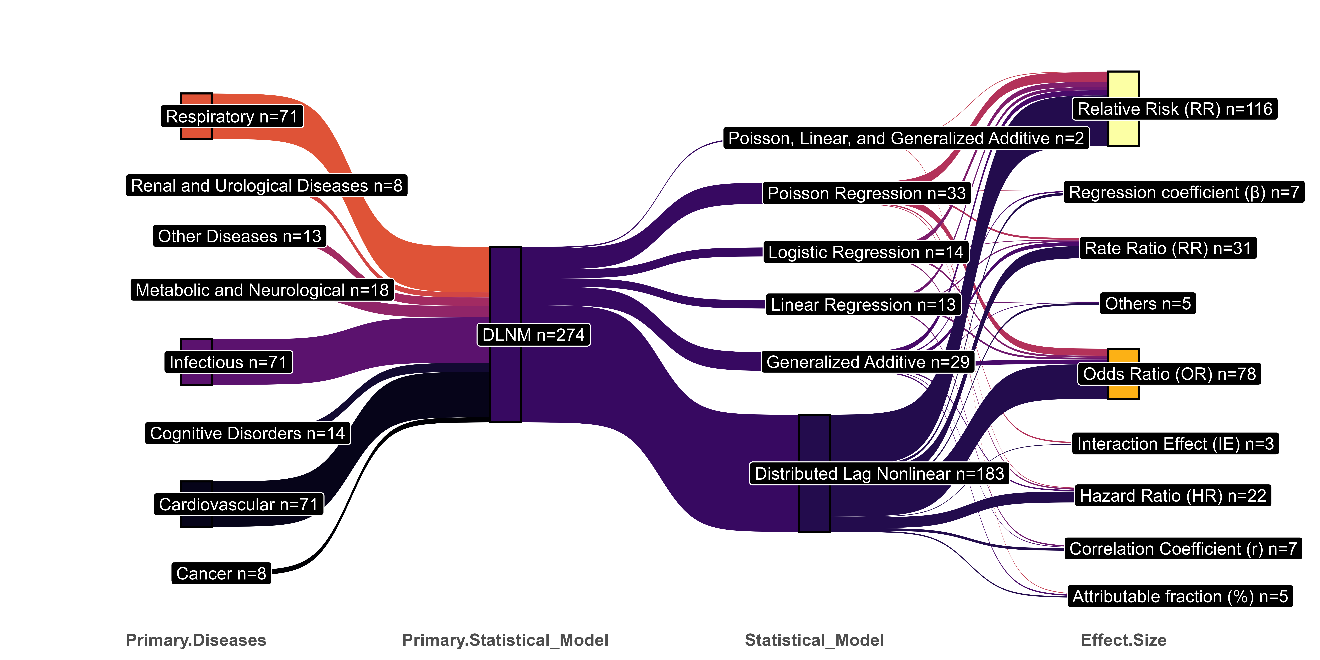


Figure S6. Snaky plot for the structural of DLNM with other Traditional Models.

Table S1. Frequency table for Sampling design, Diseases, and health outcomes.

|  | Sampling Design | | | | | | | | | | | |  |
| --- | --- | --- | --- | --- | --- | --- | --- | --- | --- | --- | --- | --- | --- |
|  | Case Crossover Study | Cohort Study | Cross-sectional study | Deep learning | Ecological study | Machine learning model | Observational study | Others | Retrospective study | Retrospective time-series study | Time-stratified case-crossover | Time Series Study | Total |
| Combined |  |  |  |  |  |  |  |  |  |  |  |  |  |
| Cancer-Incidence | 2 (0.7%) | 0 (0%) | 0 (0%) | 0 (0%) | 0 (0%) | 0 (0%) | 0 (0%) | 0 (0%) | 0 (0%) | 0 (0%) | 0 (0%) | 0 (0%) | 2(0.7%) |
| Cancer-Morbidity | 0 (0%) | 0 (0%) | 0 (0%) | 0 (0%) | 0 (0%) | 0 (0%) | 0 (0%) | 0 (0%) | 0 (0%) | 0 (0%) | 0 (0%) | 2(0.7%) | 2(0.7%) |
| Cancer - Physiological Metric | 0 (0%) | 0 (0%) | 0 (0%) | 0 (0%) | 0 (0%) | 0 (0%) | 0 (0%) | 0 (0%) | 0 (0%) | 0 (0%) | 0 (0%) | 1(0.4%) | 1(0.4%) |
| CVD &RD - Death Outcomes | 0 (0%) | 0 (0%) | 0 (0%) | 0 (0%) | 0 (0%) | 0 (0%) | 0 (0%) | 0 (0%) | 0 (0%) | 1(0.4%) | 0 (0%) | 0 (0%) | 1(0.4%) |
| CVD &RD - Morbidity | 1 (0.4%) | 1 (0.4%) | 0 (0%) | 0 (0%) | 0 (0%) | 0 (0%) | 0 (0%) | 0 (0%) | 0 (0%) | 0 (0%) | 1(0.4%) | 2 (0.7%) | 5(1.8%) |
| CVD &RD-Mortality | 2 (0.7%) | 0 (0%) | 0 (0%) | 0 (0%) | 0 (0%) | 1(0.4%) | 0 (0%) | 1(0.4%) | 0 (0%) | 0 (0%) | 0 (0%) | 2 (0.7%) | 6(2.2%) |
| CVD - Death Outcomes | 0 (0%) | 0 (0%) | 1(0.4%) | 0 (0%) | 0 (0%) | 0 (0%) | 0 (0%) | 0 (0%) | 0 (0%) | 0 (0%) | 0 (0%) | 0 (0%) | 1(0.4%) |
| CVD - Incidence | 0 (0%) | 0 (0%) | 0 (0%) | 0 (0%) | 1(0.4%) | 0 (0%) | 0 (0%) | 1(0.4%) | 0 (0%) | 0 (0%) | 0 (0%) | 1 (0.4%) | 3(1.1%) |
| CVD - Morbidity | 3 (1.1%) | 3 (1.1%) | 1(0.4%) | 0 (0%) | 1(0.4%) | 0 (0%) | 3 (1.1%) | 2(0.7%) | 2 (0.7%) | 0 (0%) | 3(1.1%) | 27(9.9%) | 45(16%) |
| CVD - Mortality | 2 (0.7%) | 1 (0.4%) | 0 (0%) | 0 (0%) | 0 (0%) | 0 (0%) | 0 (0%) | 0 (0%) | 0 (0%) | 1(0.4%) | 0 (0%) | 3 (1.1%) | 7(2.6%) |
| CVD-Physiological Metric | 0 (0%) | 0 (0%) | 0 (0%) | 0 (0%) | 0 (0%) | 0 (0%) | 0 (0%) | 0 (0%) | 0 (0%) | 0 (0%) | 0 (0%) | 1 (0.4%) | 1(0.4%) |
| CD - Death Outcomes | 1 (0.4%) | 1(0.4%) | 0 (0%) | 0 (0%) | 0 (0%) | 0 (0%) | 0 (0%) | 0 (0%) | 1 (0.4%) | 0 (0%) | 0 (0%) | 0 (0%) | 3(1.1%) |
| CD - Incidence | 0 (0%) | 0 (0%) | 0 (0%) | 0 (0%) | 1(0.4%) | 0 (0%) | 0 (0%) | 0 (0%) | 0 (0%) | 1(0.4%) | 0 (0%) | 1 (0.4%) | 3(1.1%) |
| CD - Morbidity | 0 (0%) | 1 (0.4%) | 1 (0.4%) | 0 (0%) | 0 (0%) | 0 (0%) | 0 (0%) | 1(0.4%) | 0 (0%) | 0 (0%) | 0 (0%) | 4 (1.5%) | 7(2.6%) |
| CD - Mortality | 0 (0%) | 0 (0%) | 0 (0%) | 0 (0%) | 0 (0%) | 0 (0%) | 0 (0%) | 0 (0%) | 0 (0%) | 0 (0%) | 0 (0%) | 3 (1.1%) | 3(1.1%) |
| ID - Incidence | 5 (1.8%) | 0 (0%) | 2(0.7%) | 1 (0.4%) | 2 (0.7%) | 0 (0%) | 3 (1.1%) | 5(1.8%) | 0 (0%) | 0 (0%) | 1(0.4%) | 10 (3.6%) | 29(11%) |
| ID - Infectious Outcomes | 6 (2.2%) | 1 (0.4%) | 1(0.4%) | 0 (0%) | 1 (0.4%) | 0 (0%) | 1 (0.4%) | 0 (0%) | 0 (0%) | 0 (0%) | 3(1.1%) | 16 (5.8%) | 29 (11%) |
| ID - Morbidity | 1 (0.4%) | 2 (0.7%) | 1 (0.4%) | 0 (0%) | 1 (0.4%) | 0 (0%) | 2 (0.7%) | 0 (0%) | 0 (0%) | 2(0.7%) | 0 (0%) | 3 (1.1%) | 12(4.4%) |
| ID - Mortality | 0 (0%) | 0 (0%) | 1 (0.4%) | 0 (0%) | 0 (0%) | 0 (0%) | 0 (0%) | 0 (0%) | 0 (0%) | 0 (0%) | 0 (0%) | 2 (0.7%) | 3(1.1%) |
| MND - Death Outcomes | 0 (0%) | 1 (0.4%) | 0 (0%) | 0 (0%) | 0 (0%) | 0 (0%) | 0 (0%) | 0 (0%) | 0 (0%) | 0 (0%) | 0 (0%) | 0 (0%) | 1(0.4%) |
| MND - Incidence | 0(0%) | 0(0%) | 0(0%) | 0(0%) | 0(0%) | 0(0%) | 0(0%) | 0(0%) | 0(0%) | 0(0%) | 0(0%) | 2(0.7%) | 2(0.7%) |
| MND -Morbidity | 1 (0.4%) | 1 (0.4%) | 0 (0%) | 0 (0%) | 0 (0%) | 0 (0%) | 1 (0.4%) | 2(0.7%) | 0 (0%) | 0 (0%) | 0 (0%) | 5(1.8%) | 10(3.6%) |
| MND - Mortality | 2 (0.7%) | 0 (0%) | 0 (0%) | 0 (0%) | 0 (0%) | 0 (0%) | 1 (0.4%) | 0 (0%) | 0 (0%) | 1(0.4%) | 0 (0%) | 1(0.4%) | 5(1.8%) |
| MND -Prevalence | 0 (0%) | 2 (0.7%) | 0 (0%) | 0 (0%) | 0 (0%) | 0 (0%) | 0 (0%) | 1(0.4%) | 0 (0%) | 0 (0%) | 0 (0%) | 2(0.7%) | 5(1.8%) |
| Others - Birth outcomes | 2 (0.7%) | 1(0.4%) | 0 (0%) | 0 (0%) | 0 (0%) | 0 (0%) | 0 (0%) | 0 (0%) | 0 (0%) | 0 (0%) | 0 (0%) | 0(0%) | 3(1.1%) |
| Others Morbidity | 1 (0.4%) | 0 (0%) | 0 (0%) | 0 (0%) | 0 (0%) | 0 (0%) | 1(0.4%) | 0 (0%) | 0 (0%) | 2(0.7%) | 0 (0%) | 2(0.7%) | 6(2.2%) |
| Others -Mortality | 0 (0%) | 0 (0%) | 0 (0%) | 0 (0%) | 1 (0.4%) | 0 (0%) | 0 (0%) | 0 (0%) | 0 (0%) | 0 (0%) | 0 (0%) | 1(0.4%) | 2(0.7%) |
| RUD - Morbidity | 0 (0%) | 0 (0%) | 0 (0%) | 0 (0%) | 0 (0%) | 1 (0.4%) | 0 (0%) | 0 (0%) | 0 (0%) | 0 (0%) | 0 (0%) | 6(2.2%) | 7(2.6%) |
| RUD - Mortality | 0 (0%) | 0 (0%) | 0 (0%) | 0 (0%) | 0 (0%) | 0 (0%) | 0 (0%) | 0 (0%) | 0 (0%) | 0 (0%) | 0 (0%) | 1(0.4%) | 1(0.4%) |
| RD - Death Outcomes | 0 (0%) | 0 (0%) | 0 (0%) | 0 (0%) | 0 (0%) | 1 (0.4%) | 0 (0%) | 0 (0%) | 0 (0%) | 0 (0%) | 0 (0%) | 0(0%) | 1(0.4%) |
| RD - Incidence | 1 (0.4%) | 0 (0%) | 0 (0%) | 0 (0%) | 0 (0%) | 0 (0%) | 0 (0%) | 1(0.4%) | 0 (0%) | 1(0.4%) | 0 (0%) | 4(1.5%) | 7(2.6%) |
| RD - Infectious Outcomes | 0 (0%) | 0 (0%) | 0 (0%) | 0 (0%) | 0 (0%) | 0 (0%) | 0 (0%) | 0 (0%) | 0 (0%) | 0 (0%) | 1(0.4%) | 2(0.7%) | 3(1.1%) |
| RD - Morbidity | 4 (1.5%) | 5 (1.8%) | 5 (1.8%) | 1 (0.4%) | 2 (0.7%) | 0 (0%) | 3(1.1%) | 7(2.6%) | 0 (0%) | 2 (0.7%) | 0 (0%) | 10(3.6%) | 39(14%) |
| RD - Mortality | 0 (0%) | 1 (0.4%) | 0 (0%) | 0 (0%) | 0 (0%) | 0 (0%) | 2(0.7%) | 0 (0%) | 0 (0%) | 0 (0%) | 1(0.4%) | 11(4.0%) | 15(5.5%) |
| RD - Physiological Metric | 0 (0%) | 0 (0%) | 0 (0%) | 0 (0%) | 0 (0%) | 0 (0%) | 0 (0%) | 0 (0%) | 0 (0%) | 0 (0%) | 0 (0%) | 1(0.4%) | 1(0.4%) |
| RD - Prevalence | 0 (0%) | 0 (0%) | 0 (0%) | 0 (0%) | 0 (0%) | 0 (0%) | 0 (0%) | 0 (0%) | 0 (0%) | 0 (0%) | 0 (0%) | 1(0.4%) | 1(0.4%) |
| RID - Death Outcomes | 0 (0%) | 0 (0%) | 0 (0%) | 0 (0%) | 0 (0%) | 0 (0%) | 0 (0%) | 0 (0%) | 0 (0%) | 0 (0%) | 0 (0%) | 1(0.4%) | 1(0.4%) |
| RID - Incidence | 0 (0%) | 0 (0%) | 0 (0%) | 0 (0%) | 0 (0%) | 0 (0%) | 0 (0%) | 0 (0%) | 0 (0%) | 1(0.4%) | 0 (0%) | 0(0%) | 1(0.4%) |
| Total | 34 (12%) | 21 (7.7%) | 13 (4.7%) | 2 (0.7%) | 10 (3.6%) | 3 (1.1%) | 17(6.2%) | 21(7.7%) | 3(1.1%) | 12(4.4%) | 10 (3.6%) | 128(47%) | 274(100%) |

Table S2. Frequency table for Sampling design, Countries, and health outcomes.

|  | Sampling Design | | | | | | | | | | | |  |
| --- | --- | --- | --- | --- | --- | --- | --- | --- | --- | --- | --- | --- | --- |
|  | Case Crossover Study | Cohort Study | Cross-sectional study | Deep learning | Ecological study | Machine learning model | Observational study | Others | Retrospective study | Retrospective time-series study | Time-stratified case-crossover | Time Series Study | Total |
| Combined |  |  |  |  |  |  |  |  |  |  |  |  |  |
| Australia - Morbidity | 0 (0%) | 0 (0%) | 0 (0%) | 0 (0%) | 0 (0%) | 0 (0%) | 0 (0%) | 0 (0%) | 0 (0%) | 0 (0%) | 0 (0%) | 3 (1.1%) | 3 (1.1%) |
| China - Birth outcomes | 2 (0.7%) | 1 (0.4%) | 0 (0%) | 0 (0%) | 0 (0%) | 0 (0%) | 0 (0%) | 0 (0%) | 0 (0%) | 0 (0%) | 0 (0%) | 0 (0%) | 3 (1.1%) |
| China - Death Outcomes | 1 (0.4%) | 2 (0.7%) | 0 (0%) | 0 (0%) | 0 (0%) | 1 (0.4%) | 0 (0%) | 0 (0%) | 0 (0%) | 0 (0%) | 0 (0%) | 0 (0%) | 4 (1.5%) |
| China - Incidence | 3(1.1%) | 0(0%) | 1(0.4%) | 0(0%) | 4(1.5%) | 0(0%) | 1 (0.4%) | 2(0.7%) | 0 (0%) | 2 (0.7%) | 1(0.4%) | 9(3.3%) | 23(8.4%) |
| China - Infectious Outcomes | 4(1.5%) | 1(0.4%) | 0(0%) | 0(0%) | 0 (0%) | 0(0%) | 0 (0%) | 0(0%) | 0 (0%) | 0 (0%) | 3(1.1%) | 14(5.1%) | 22(8.0%) |
| China - Morbidity | 7(2.6%) | 9(3.3%) | 6(2.2%) | 1(0.4%) | 3(1.1%) | 1(0.4%) | 7 (2.6%) | 8(2.9%) | 1 (0.4%) | 2 (0.7%) | 2(0.7%) | 36(13%) | 83(30%) |
| China - Mortality | 4(1.5%) | 1(0.4%) | 0(0%) | 0(0%) | 0 (0%) | 1(0.4%) | 2(0.7%) | 1(0.4%) | 0 (0%) | 2 (0.7%) | 1(0.4%) | 12(4.4%) | 24(8.8%) |
| China - Physiological Metric | 0 (0%) | 0(0%) | 0(0%) | 0(0%) | 0 (0%) | 0(0%) | 0 (0%) | 0(0%) | 0 (0%) | 0 (0%) | 0 (0%) | 1(0.4%) | 1(0.4%) |
| China - Prevalence | 0 (0%) | 1(0.4%) | 0(0%) | 0(0%) | 0 (0%) | 0(0%) | 0 (0%) | 1(0.4%) | 0 (0%) | 0 (0%) | 0 (0%) | 3(1.1%) | 5(1.8%) |
| Europe - Incidence | 2 (0.7%) | 0 (0%) | 0 (0%) | 0(0%) | 0 (0%) | 0(0%) | 1 (0.4%) | 0(0%) | 0 (0%) | 0 (0%) | 0 (0%) | 2(0.7%) | 5(1.8%) |
| Europe - Infectious Outcomes | 1(0.4%) | 0(0%) | 0(0%) | 0(0%) | 0 (0%) | 0(0%) | 1 (0.4%) | 0(0%) | 0 (0%) | 0 (0%) | 1(0.4%) | 0(0%) | 3(1.1%) |
| Europe - Morbidity | 1(0.4%) | 0(0%) | 0(0%) | 0(0%) | 0 (0%) | 0(0%) | 1 (0.4%) | 2(0.7%) | 1(0.4%) | 0 (0%) | 2(0.7%) | 7(2.6%) | 14(5.1%) |
| Europe - Mortality | 1(0.4%) | 0(0%) | 0(0%) | 0(0%) | 0 (0%) | 0(0%) | 0 (0%) | 0(0%) | 0 (0%) | 0 (0%) | 0 (0%) | 3(1.1%) | 4(1.5%) |
| Global - Incidence | 1(0.4%) | 0(0%) | 0(0%) | 0(0%) | 0 (0%) | 0(0%) | 0 (0%) | 1(0.4%) | 0 (0%) | 0 (0%) | 0 (0%) | 0(0%) | 2(0.7%) |
| Global - Infectious Outcomes | 0 (0%) | 0(0%) | 1(0.4%) | 0(0%) | 1(0.4%) | 0(0%) | 0 (0%) | 0(0%) | 0 (0%) | 0 (0%) | 0 (0%) | 0(0%) | 2(0.7%) |
| Global - Morbidity | 1(0.4%) | 0(0%) | 0(0%) | 0(0%) | 0 (0%) | 0(0%) | 0 (0%) | 0(0%) | 0 (0%) | 1 (0.4%) | 0 (0%) | 0(0%) | 2(0.7%) |
| Global - Mortality | 0 (0%) | 0(0%) | 0(0%) | 0(0%) | 0 (0%) | 0(0%) | 0 (0%) | 0(0%) | 0 (0%) | 0 (0%) | 0(0%) | 1(0.4%) | 1(0.4%) |
| North America - Incidence | 1(0.4%) | 0(0%) | 0(0%) | 0(0%) | 0 (0%) | 0(0%) | 0 (0%) | 1(0.4%) | 0 (0%) | 0 (0%) | 0 (0%) | 3(1.1%) | 5(1.8%) |
| North America - Infectious Outcomes | 0 (0%) | 0(0%) | 0(0%) | 0(0%) | 0 (0%) | 0(0%) | 0 (0%) | 0(0%) | 0(0%) | 0 (0%) | 0 (0%) | 2(0.7%) | 2(0.7%) |
| North America - Morbidity | 1(0.4%) | 2(0.7%) | 2(0.7%) | 0(0%) | 1(0.4%) | 0(0%) | 2(0.7%) | 1(0.4%) | 0 (0%) | 2 (0.7%) | 0 (0%) | 2(0.7%) | 13(4.7%) |
| North America - Mortality | 0 (0%) | 0(0%) | 0(0%) | 0(0%) | 0 (0%) | 0(0%) | 1(0.4%) | 0(0%) | 0 (0%) | 0 (0%) | 0 (0%) | 2(0.7%) | 3(1.1%) |
| Other Asian - Death Outcomes | 0 (0%) | 0(0%) | 1(0.4%) | 0(0%) | 0 (0%) | 0(0%) | 0(0%) | 0(0%) | 1 (0.4%) | 1 (0.4%) | 0 (0%) | 1(0.4%) | 4(1.5%) |
| Other Asian - Incidence | 1(0.4%) | 0(0%) | 1(0.4%) | 1(0.4%) | 0 (0%) | 0(0%) | 1(0.4%) | 3(1.1%) | 0 (0%) | 1(0.4%) | 0 (0%) | 4(1.5%) | 12(4.4%) |
| Other Asian - Infectious Outcomes | 1 (0.4%) | 0(0%) | 0(0%) | 0(0%) | 0 (0%) | 0(0%) | 0 (0%) | 0(0%) | 0 (0%) | 0 (0%) | 0 (0%) | 2(0.7%) | 3(1.1%) |
| Other Asian - Morbidity | 1(0.4%) | 2(0.7%) | 0(0%) | 0(0%) | 0 (0%) | 0(0%) | 0 (0%) | 1(0.4%) | 0(0%) | 1(0.4%) | 0 (0%) | 13(4.7%) | 18(6.6%) |
| Other Asian - Mortality | 1(0.4%) | 1(0.4%) | 1(0.4%) | 0(0%) | 1(0.4%) | 0 (0%) | 0 (0%) | 0(0%) | 0 (0%) | 0 (0%) | 0 (0%) | 6(2.2%) | 10(3.6%) |
| Other Asian - Physiological Metric | 0 (0%) | 0(0%) | 0(0%) | 0(0%) | 0 (0%) | 0(0%) | 0 (0%) | 0(0%) | 0 (0%) | 0 (0%) | 0 (0%) | 2(0.7%) | 2(0.7%) |
| Other Asian - Prevalence | 0 (0%) | 1(0.4%) | 0(0%) | 0(0%) | 0 (0%) | 0(0%) | 0 (0%) | 0(0%) | 0 (0%) | 0 (0%) | 0 (0%) | 0(0%) | 1(0.4%) |
| Total | 34(12%) | 21(7.7%) | 13(4.7%) | 2(0.7%) | 10(3.6%) | 3(1.1%) | 17(6.2%) | 21(7.7%) | 3 (1.1%) | 12(4.4%) | 10(3.6%) | 128(47%) | 274(100%) |

Table S3. Descriptive summary of DLNM studies focused on cardiovascular diseases (CVDs), showing associations between study outcomes, geographic locations, study designs, and environmental exposure variables.

| Diseases | Response Variables | | Region | Location | Study Plan | Exposure Variables |
| --- | --- | --- | --- | --- | --- | --- |
| Acute Myocardial Infarction (AMI) | | Hospital Admissions | China | China | Retrospective study | Air Pollutant Factors |
| Acute Myocardial Infarction (AMI) | | Hospitalisation | Europe | Spain | Time-stratified case-crossover | Temperature Factors |
| Acute Myocardial Infarction (AMI) | | Hospitalisation | China | China | Time Series Study | Temperature Factors |
| Acute Myocardial Infarction (AMI) | | Hospitalisation | China | China | Time Series Study | Air Pollutant Factors |
| Aortic diseases | | Hospitalisation | North America | Brazil | Observational study | Temperature Factors |
| Arrhythmias | | Hospitalisation | China | China | Time Series Study | Air Pollutant Factors |
| Hypertension | | Hospital Admissions | China | China | Time-stratified case-crossover | Temperature Factors |
| Cardiovascular disease (CVD) | | Outpatient visits | Asian Countries | South Korea | Others | Temperature Factors |
| Cardiovascular and Respiratory Diseases | | Hospital Admissions | North America | Brazil | Time Series Study | Heat Waves |
| Cardiovascular and Respiratory Diseases | | Hospital Admissions | China | China | Cohort Study | Air Pollutant Factors |
| Cardiovascular and Respiratory Diseases | | Hospital Admissions | China | China | Ecological study | Air Pollutant Factors |
| Cardiovascular and Respiratory Diseases | | Hospital Admissions | Asian Countries | Singapore | Case Crossover Study | Air Pollutant Factors |
| Cardiovascular and Respiratory Diseases | | Hospitalisation | Europe | Sweden | Time Series Study | Temperature Factors |
| Cardiovascular disease (CVD) | | Ambulance Calls | Asian Countries | Taiwan | Time Series Study | Temperature Factors |
| Cardiovascular disease (CVD) | | Death Outcomes | Europe | UK | Cross-sectional study | Temperature Factors |
| Cardiovascular disease (CVD) | | Emergency room visits | Europe | Belgium | Case Crossover Study | Temperature Factors |
| Cardiovascular disease (CVD) | | Emergency room visits | China | China | Time Series Study | Temperature Factors |
| Cardiovascular disease (CVD) | | Emergency room visits | Asian Countries | Malaysia | Retrospective study | Temperature /Humidity |
| Cardiovascular disease (CVD) | | Physiological Metric | China | China | Time Series Study | Temperature Factors |
| Cardiovascular disease (CVD) | | Hospital Admissions | China | China | Time Series Study | Temperature Factors |
| Cardiovascular disease (CVD) | | Hospital Admissions | Asian Countries | Iran | Time Series Study | Temperature Factors |
| Cardiovascular disease (CVD) | | Hospital Admissions | China | China | Time Series Study | Air Pollutant Factors |
| Cardiovascular disease (CVD) | | Hospital Admissions | China | China | Time Series Study | Temperature Factors |
| Cardiovascular disease (CVD) | | Hospital Admissions | China | China | Cross-sectional study | Temperature Factors |
| Cardiovascular disease (CVD) | | Hospital Admissions | China | China | Time Series Study | Temperature Factors |
| Cardiovascular disease (CVD) | | Hospital Admissions | China | China | Time Series Study | Air Pollutant Factors |
| Cardiovascular disease (CVD) | | Hospital Admissions | China | China | Observational study | Temperature Factors |
| Cardiovascular disease (CVD) | | Hospital Admissions | China | China | Time Series Study | Temperature Factors |
| Cardiovascular disease (CVD) | | Hospital Admissions | China | China | Time Series Study | Air Pollutant Factors |
| Cardiovascular disease (CVD) | | Hospital Admissions | China | China | Time Series Study | Metrological Factors |
| Cardiovascular disease (CVD) | | Hospital Admissions | China | China | Time-stratified case-crossover | Air Pollutant Factors |
| Cardiovascular disease (CVD) | | Hospital Admissions | China | China | Time Series Study | Air Pollutant Factors |
| Cardiovascular disease (CVD) | | Hospital Admissions | China | China | Time Series Study | Temperature Factors |
| Cardiovascular disease (CVD) | | Hospital Admissions | China | China | Time Series Study | Temperature Factors |
| Cardiovascular disease (CVD) | | Hospital Admissions and Deaths | China | China | Time Series Study | Temperature Factors |
| Cardiovascular disease (CVD) | | Hospitalisation | China | China | Others | Temperature Factors |
| Cardiovascular disease (CVD) | | Hospitalisation | Europe | Italy | Time Series Study | Air Pollutant Factors |
| Cardiovascular disease (CVD) | | Hospitalisation | Asian Countries | Japan | Observational study | Air Pollutant Factors |
| Cardiovascular disease (CVD) | | Incidence | China | China | Time Series Study | Temperature Factors |
| Cardiovascular disease (CVD) | | Hospitalisation | China | China | Time Series Study | Temperature Factors |
| Cardiovascular disease (CVD) | | Mortality | China | China | Time Series Study | Temperature Factors |
| Cardiovascular disease (CVD) | | Mortality | China | China | Cohort Study | Air Pollutant Factors |
| Cardiovascular disease (CVD) | | Mortality | China | China | Case Crossover Study | Air Pollutant Factors |
| Cardiovascular disease (CVD) | | Outpatient visits | Asian Countries | Iran | Time Series Study | Temperature Factors |
| Cardiovascular disease (CVD) | | Outpatient visits | Europe | Cyprus | Case Crossover Study | Temperature Factors |
| Coronary artery disease (CAD) | | Hospital Admissions | China | China | Time Series Study | Air Pollutant Factors |
| Coronary Heart Disease (CHD) | | Hospital Admissions | China | China | Time Series Study | Air Pollutant Factors |
| Death Outcomes | | Mortality | Asian Countries | Bangladesh | Retrospective time-series study | Temperature Factors |
| Heatstroke | | Incidence | China | China | Ecological study | Air Pollutant Factors |
| Hypertension | | Hospital visits | China | China | Time Series Study | Metrological Factors |
| Hypertension | | Hospital Admissions | China | China | Cohort Study | Air Pollutant Factors |
| Hypertension | | Hospital Admissions | China | China | Time Series Study | Temperature Factors |
| Ischemic Stroke | | Mortality | China | China | Time Series Study | Air Pollutant Factors |
| Ischemic Stroke | | Mortality | Europe | Spain | Time Series Study | Air Pollutant Factors |
| Stroke | | Hospitalisation | China | China | Cohort Study | Temperature Factors |
| Stroke | | Hospitalisation | China | China | Time Series Study | Air Pollutant Factors |
| Stroke | | Incidence | Global | Global | Others | Temperature Factors |
| Stroke | | Inpatient visits | China | China | Case Crossover Study | Air Pollutant Factors |
| Cardiovascular disease (CVD) | | Emergency room visits | China | China | Time Series Study | Air Pollutant Factors |
| Cardiovascular and Respiratory Diseases | | Death Outcomes | China | China | Time Series Study | Temperature Factors |
| Cardiovascular and Respiratory Diseases | | Ambulance Calls | Europe | Greece | Time-stratified case-crossover | Temperature Factors |
| Cardiovascular and Respiratory Diseases | | Ambulance Calls | Other Asian Countries | Taiwan | Case Crossover Study | Air Pollutant Factors |
| Cardiovascular and Respiratory Diseases | | Death Outcomes | China | China | Retrospective time-series study | Temperature Factors |
| Cardiovascular and Respiratory Diseases | | Emergency room visits | North America | USA | Time Series Study | Heat Waves |
| Cardiovascular and Respiratory Diseases | | Mortality | Asian Countries | Iran | Others | Temperature Factors |
| Cardiovascular and Respiratory Diseases | | Mortality | China | China | Machine learning model | Temperature Factors |
| Cardiovascular and Respiratory Diseases | | Mortality | China | China | Time Series Study | Temperature Factors |
| Cardiovascular and Respiratory Diseases | | Mortality | Asian Countries | Iran | Case Crossover Study | Air Pollutant Factors |
| Cardiovascular and Respiratory Diseases | | Mortality | North America | Brazil | Time Series Study | Temperature Factors |
| Cardiovascular and Respiratory Diseases | | Mortality | China | China | Case Crossover Study | Temperature Factors |
| Cardiovascular and Respiratory Diseases | | Outpatient visits | Asian Countries | Japan | Cohort Study | Air Pollutant Factors |

Table S4. Frequency table Across the RDs characteristics.

| Response Variables | Diseases | Region | Location | Study Plan | Exposure Variables |
| --- | --- | --- | --- | --- | --- |
| Emergency room visits | Asthma | China | China | Cross-sectional study | Air Pollutant /Temperature |
| Hospital visits | Asthma | Europe | Poland | Time Series Study | Temperature Factors |
| Hospital visits | Asthma | Asian Country | South Korea | Cohort Study | Air Pollutant Factors |
| Hospital visits | Asthma | Europe | Sweden | Time Series Study | Temperature /Air Quality |
| Hospital visits | Asthma | North America | USA | Observational study | Air Pollutant Factors |
| Hospitalisation | Asthma | China | China | Cross-sectional study | Air Pollutant Factors |
| Hospitalisation | Asthma | China | China | Cohort Study | Air Pollutant /Temperature |
| Hospitalisation | Asthma | North America | Mexico | Retrospective time-series study | Heavy Metals |
| Incidence | Asthma | Asian Country | Taiwan | Case Crossover Study | Meteorological Factors |
| Hospital Admissions | Asthma | China | China | Cohort Study | Air Pollutant Factors |
| Hospital Admissions | Asthma | China | China | Time-stratified case-crossover | Air Pollutant Factors |
| Hospital Admissions | Childhood asthma | China | China | Case Crossover Study | Temperature Factors |
| Hospital visits | Childhood asthma | China | China | Time Series Study | Temperature Factors |
| Hospitalisation | Childhood asthma | China | China | Ecological study | Temperature Factors |
| Outpatient visits | Childhood asthma | China | China | Others | Air Pollutant Factors |
| Outpatient visits | Childhood asthma | China | China | Others | Temperature Factors |
| Hospital Admissions | COPD | China | China | Others | Air Pollutant Factors |
| Hospitalization | COPD | North America | USA | Ecological study | Air Pollutant Factors |
| Hospitalisation | COPD | Europe | Serbia | Others | Air Pollutant Factors |
| Hospitalisation | Lung function | Europe | Kenya | Time Series Study | Air Pollutant Factors |
| Ambulance Calls | Respiratory Disease (RD) | China | China | Case Crossover Study | Air Pollutant Factors |
| Death Outcomes | Respiratory Disease (RD) | China | China | Machine learning model | Temperature Factors |
| Physiological Metric | Respiratory Disease (RD) | Asian Country | Thailand | Time Series Study | Air Pollutant Factors |
| Emergency room visits | Respiratory Disease (RD) | China | China | Cohort Study | Meteorological Factors |
| Emergency room visits | Respiratory Disease (RD) | China | China | Observational study | Temperature /Air Quality |
| Emergency room visits | Respiratory Disease (RD) | Asian Country | Malaysia | Time Series Study | Temperature Factors |
| Emergency room visits | Respiratory Disease (RD) | Asian Country | South Africa | Time Series Study | Air Pollutant /Temperature |
| Emergency room visits | Respiratory Disease (RD) | China | China | Deep learning | Air Pollutant Factors |
| Emergency room visits | Respiratory Disease (RD) | Europe | Spain | Case Crossover Study | Air Pollutant Factors |
| Emergency room visits | Respiratory Disease (RD) | China | China | Cross-sectional study | Air Pollutant Factors |
| Emergency room visits | Respiratory Disease (RD) | China | China | Time Series Study | Temperature Factors |
| Hospital Admissions | Respiratory Disease (RD) | China | China | Time Series Study | Temperature Factors |
| Hospital Admissions | Respiratory Disease (RD) | Asian Country | South Korea | Time Series Study | Air Pollutant /Temperature |
| Hospital Admissions | Respiratory Disease (RD) | Europe | Poland | Time Series Study | Temperature Factors |
| Hospital visits | Respiratory Disease (RD) | China | China | Time Series Study | Air Pollutant Factors |
| Hospital visits | Respiratory Disease (RD) | China | China | Time Series Study | Air Pollutant Factors |
| Hospitalisation | Respiratory Disease (RD) | North America | Brazil | Retrospective time-series study | Temperature Factors |
| Hospitalisation | Respiratory Disease (RD) | Asian Country | Taiwan | Time Series Study | Air Pollutant Factors |
| Hospitalisation | Respiratory Disease (RD) | North America | USA | Others | Temperature Factors |
| Hospitalisation | Respiratory Disease (RD) | China | China | Case Crossover Study | Temperature Factors |
| Hospitalisation | Respiratory Disease (RD) | China | China | Observational study | Air Pollutant Factors |
| Hospitalisation | Respiratory Disease (RD) | China | China | Cross-sectional study | Air Pollutant/Temperature |
| Hospitalisation | Respiratory Disease (RD) | China | China | Cohort Study | Temperature /Humidity |
| Hospitalisation | Respiratory Disease (RD) | Europe | France | Time Series Study | Temperature Factors |
| Hospitalisation | Respiratory Disease (RD) | North America | USA | Cross-sectional study | Air Pollutant/Temperature |
| Hospitalisation | Respiratory Disease (RD) | China | China | Observational study | Temperature Factors |
| Incidence | Respiratory Disease (RD) | China | China | Others | Temperature /Humidity |
| Incidence | Respiratory Disease (RD) | China | China | Retrospective time-series study | Air Pollutant /Temperature |
| Incidence | Respiratory Disease (RD) | Asian Country | Taiwan | Time Series Study | Air Pollutant Factors |
| Incidence | Respiratory Disease (RD) | China | China | Time Series Study | Air Pollutant Factors |
| Incidence | Respiratory Disease (RD) | China | China | Time Series Study | Temperature Factors |
| Infecetion Cases | Respiratory Disease (RD) | China | China | Time-stratified case-crossover | Meteorological Factors |
| Infecetion Cases | Respiratory Disease (RD) | North America | Mexico | Time Series Study | Temperature Factors |
| Infection Cases | Respiratory Disease (RD) | China | China | Time Series Study | Air Pollutant Factors |
| Inpatient visits | Respiratory Disease (RD) | China | China | Time Series Study | Temperature Factors |
| Mortality | Respiratory Disease (RD) | Asian Country | Iran | Time Series Study | Temperature Factors |
| Mortality | Respiratory Disease (RD) | Asian Country | Japan | Time Series Study | Air Pollutant Factors/Humidity |
| Mortality | Respiratory Disease (RD) | China | China | Time Series Study | Air Pollutant Factors |
| Mortality | Respiratory Disease (RD) | China | China | Observational study | Air Pollutant Factors |
| Mortality | Respiratory Disease (RD) | China | China | Time Series Study | Temperature Factors |
| Outpatient visits | Respiratory Disease (RD) | China | China | Others | Temperature Factors |
| Outpatient visits | Respiratory Disease (RD) | China | China | Others | Air Pollutant Factors |
| Outpatient visits | Respiratory Disease (RD) | China | China | Cohort Study | Temperature Factors |
| Outpatient visits | Respiratory Disease (RD) | Asian Country | Japan | Cohort Study | Air Pollutant Factors |
| Prevalence | Respiratory Disease (RD) | China | China | Time Series Study | Air Pollutant Factors |
| Hospital visits | Respiratory infectious diseases | China | China | Time Series Study | Temperature Factors |
| Hospital visits | Respiratory infectious diseases | China | China | Time-stratified case-crossover | Air Pollutant Factors |
| Death Outcomes | Respiratory infectious diseases | Asian Country | Iran | Time Series Study | Air Pollutant Factors |
| Incidence | Respiratory infectious diseases | China | China | Retrospective time-series study | Air Pollutant Factors |
| Hospitalisation | Bronchitis | China | China | Time Series Study | Air Pollutant Factors |
| Incidence | Chickenpox (varicella) | China | China | Time Series Study | Meteorological Factors |

Table S5. Frequency table Across the IDs characteristics.

| Response Variables | Diseases | Region | Location | Study Plan | Exposure Variables |
| --- | --- | --- | --- | --- | --- |
| Incidence | Brucellosis | Europe | Greece | Case Crossover Study | Meteorological Factors |
| Hospitalisation | COVID-19 | Global | Global | Retrospective time-series study | Environmental Factors |
| Hospitalisation | COVID-19 | Asian Countries | Bangladesh | Retrospective time-series study | Temperature Factors |
| Incidence | COVID-19 | North America | USA | Time Series Study | Temperature Factors |
| Incidence | COVID-19 | North America | USA | Others | Temperature Factors |
| Incidence | COVID-19 | North America | Canada | Time Series Study | Temperature/Humidity |
| Infecetion Cases | COVID-19 | Global | Global | Cross-sectional study | Temperature Factors |
| Infecetion Cases | COVID-19 | Global | Global | Ecological study | Temperature Factors |
| Infecetion Cases | COVID-19 | China | China | Time Series Study | Temperature/Humidity |
| Infecetion Cases | COVID-19 | China | China | Cohort Study | Temperature Factors |
| Infecetion Cases | COVID-19 | China | China | Time Series Study | Temperature /Humidity |
| Infecetion Cases | COVID-19 | China | China | Time Series Study | Temperature Factors |
| Infecetion Cases | COVID-19 | Europe | UK | Time-stratified case-crossover | Meteorological Factors |
| Infecetion Cases | COVID-19 | Asian Countries | Bangladesh | Time Series Study | Temperature Factors |
| Infecetion Cases | COVID-19 | North America | USA | Time Series Study | Temperature Factors |
| Mortality | COVID-19 | Asian Countries | Japan | Cross-sectional study | Meteorological Factors |
| Mortality | COVID-19 | Asian Countries | South Korea | Time Series Study | Temperature Factors |
| Emergency room visits | Dengue fever | China | China | Time Series Study | Meteorological/Air Pollutants Factors |
| Incidence | Dengue fever | Asian Countries | Singapore | Others | Temperature Factors |
| Incidence | Dengue fever | Asian Countries | Taiwan | Others | Temperature Factors |
| Incidence | Dengue fever | Europe | Lao PDR | Case Crossover Study | Temperature Factors |
| Incidence | Dengue fever | Asian Countries | Taiwan | Time Series Study | Environmental Factors |
| Incidence | Diarrheal diseases | Asian Countries | Taiwan | Others | Meteorological Factors |
| Infecetion Cases | Diarrheal diseases | China | China | Time Series Study | Meteorological Factors |
| Infecetion Cases | HFMD | China | China | Time Series Study | Meteorological Factors |
| Infecetion Cases | HFMD | China | China | Time Series Study | Air Pollutants Factors |
| Infecetion Cases | HFMD | China | China | Time Series Study | Temperature Factors |
| Hospitalisation | Infection Diseases | North America | Brazil | Case Crossover Study | Meteorological Factors |
| Infecetion Cases | Infection Diseases | China | China | Time Series Study | Meteorological Factors |
| Infecetion Cases | Infection Diseases | Europe | Finland | Observational study | Air Pollutants Factors |
| Infecetion Cases | Infection Diseases | China | China | Case Crossover Study | Temperature Factors |
| Infecetion Cases | Infection Diseases | China | China | Case Crossover Study | Temperature Factors |
| Hospital Admissions | Influenza | China | China | Ecological study | Meteorological Factors |
| Incidence | Influenza | China | China | Ecological study | Temperature Factors |
| Incidence | Influenza | China | China | Cross-sectional study | Temperature Factors |
| Incidence | Influenza | China | China | Time Series Study | Temperature Factors |
| Incidence | Influenza | China | China | Observational study | Temperature Factors |
| Incidence | Influenza | China | China | Time-stratified case-crossover | Environmental Factors |
| Incidence | Influenza | Europe | Macau | Observational study | Meteorological Factors |
| Incidence | Influenza | Europe | Poland | Observational study | Air Pollutants Factors |
| Incidence | Influenza | China | China | Case Crossover Study | Air Pollutants Factors |
| Incidence | Influenza | China | China | Ecological study | Air Pollutants Factors |
| Incidence | Influenza | China | China | Case Crossover Study | Temperature Factors |
| Incidence | Influenza | North America | USA | Time Series Study | Air Pollutants Factors |
| Infecetion Cases | Influenza | China | China | Time Series Study | Temperature /Humidity |
| Infecetion Cases | Influenza | China | China | Time-stratified case-crossover | Temperature Factors |
| Infecetion Cases | Influenza | China | China | Case Crossover Study | Temperature Factors |
| Infecetion Cases | Influenza | China | China | Time Series Study | Meteorological Factors |
| Infecetion Cases | Influenza | China | China | Time Series Study | Air Pollutants Factors |
| Infecetion Cases | Influenza | Europe | Spain | Case Crossover Study | Air Pollutants Factors |
| Infecetion Cases | Influenza | China | China | Time-stratified case-crossover | Air Pollutants Factors |
| Incidence | Malaria | Europe | Uganda | Time Series Study | Temperature Factors |
| Mortality | Pneumonia | Global | Global | Time Series Study | Temperature Factors |
| Outpatient visits | Pneumonia | China | China | Cohort Study | Air Pollutants Factors |
| Infecetion Cases | SARS-CoV-2 | Asian Countries | Japan | Time Series Study | Temperature Factors |
| Infecetion Cases | Scarlet fever | China | China | Time Series Study | Meteorological Factors |
| Infecetion Cases | Scarlet fever | China | China | Time Series Study | Meteorological Factors |
| Infecetion Cases | Scarlet fever | Asian Countries | Hong Kong | Case Crossover Study | Air Pollutants Factors |
| Incidence | Tuberculosis (TB) | China | China | Time Series Study | Temperature Factors |
| Incidence | Tuberculosis (TB) | China | China | Time Series Study | Temperature Factors |
| Incidence | Tuberculosis (TB) | Asian Countries | Hong Kong | Deep learning | Meteorological Factors |
| Incidence | Tuberculosis (TB) | Asian Countries | Hong Kong | Time Series Study | Air Pollutants Factors |
| Incidence | Tuberculosis (TB) | Asian Countries | Japan | Cross-sectional study | Air Pollutants Factors |
| Incidence | Tuberculosis (TB) | China | China | Case Crossover Study | Meteorological Factors |
| Incidence | Tuberculosis (TB) | China | China | Others | Temperature Factors |
| Outpatient visits | Tuberculosis (TB) | China | China | Cross-sectional study | Air Pollutants Factors |
| Outpatient visits | Tuberculosis (TB) | China | China | Observational study | Air Pollutants Factors |
| Outpatient visits | Tuberculosis (TB) | China | China | Time Series Study | Air Pollutants Factors |
| Outpatient visits | Tuberculosis (TB) | China | China | Observational study | Air Pollutants Factors |
| Hospitalisation | Viral Infectious Disease | Europe | Macao | Time Series Study | Meteorological Factors |
| Infecetion Cases | Viral Infectious Disease | China | China | Case Crossover Study | Temperature Factors |

Table S6. Frequency table Across the Metabolic, Neurological and other diseases characteristics.

| Response Variables | Diseases | Region | Location | Study Plan | Exposure Variables |
| --- | --- | --- | --- | --- | --- |
| Death Outcomes | Dementia | China | China | Cohort Study | Meteorological/Temperature |
| Mortality | Dementia | China | China | Retrospective time-series study | Heat Waves |
| Clinic Visits | Epilepsy | China | China | Observational study | Air Pollutant Factors |
| Ambulance Calls | Gestational diabetes | North America | Canada | Observational study | Meteorological factors |
| Incidence | Gestational diabetes | China | China | Time Series Study | Meteorological factors |
| Prevalence | Gestational diabetes | China | China | Time Series Study | Air Pollutant Factors |
| Prevalence | Gestational diabetes | China | China | Others | Temperature Factors |
| Prevalence | Gestational diabetes | China | China | Time Series Study | Temperature Factors |
| Prevalence | Gestational diabetes | China | China | Cohort Study | Air Pollutant Factors |
| Prevalence | Gestational diabetes | Asian countries | Japan | Cohort Study | Air Pollutant Factors |
| Emergency room visits | Ischemic Stroke | China | China | Time Series Study | Air Pollutant Factors |
| Emergency room visits | Ischemic Stroke | Australia | Australia | Time Series Study | Air Pollutant Factors |
| Hospital Admissions | Ischemic Stroke | China | China | Time Series Study | Temperature /Humidity |
| Hospital healthcare costs | Ischemic Stroke | Australia | Australia | Time Series Study | Temperature Factors |
| Hospital visits | Ischemic Stroke | Asian countries | Taiwan | Time Series Study | Heat Waves |
| Hospitalisation | Ischemic Stroke | Asian countries | Vietnam | Cohort Study | Temperature Factors |
| Hospitalisation | Ischemic Stroke | Europe | Belgium | Time Series Study | Temperature Factors |
| Morbidity | Ischemic Stroke | China | China | Others | Air Pollutant Factors |
| Morbidity | Ischemic Stroke | China | China | Case Crossover Study | Temperature Factors |
| Morbidity | Ischemic Stroke | Europe | Sweden | Others | Temperature Factors |
| Mortality | Mental disorders | Europe | Belgium | Case Crossover Study | Temperature Factors |
| medical records | Metabolic Syndrome | China | China | Case Crossover Study | Temperature Factors |
| Incidence | Seizures | China | China | Time Series Study | Temperature Factors |
| Mortality | Death Outcomes | Asian countries | Japan | Ecological study | Temperature /Humidity |
| Hospitalisation | Gout | China | China | Retrospective time-series study | Air Pollutant Factors |
| Morbidity | Kawasaki Disease | Global | Global | Case Crossover Study | Temperature Factors |
| Morbidity | Kidney Disease | Asian countries | Japan | Time Series Study | Air Pollutant Factors |
| Mortality | Kidney Disease | North America | USA | Time Series Study | Air Pollutant Factors |
| Birth outcomes | Preterm birth (PTB) | Europe | Poland | Cohort Study | Air Pollutant Factors |
| Birth outcomes | Preterm birth (PTB) | China | China | Case Crossover Study | Temperature Factors |
| Birth outcomes | Preterm birth (PTB) | China | China | Case Crossover Study | Temperature Factors |
| Emergency room visits | Various diseases | North America | USA | Observational study | Temperature Factors |
| Emergency room visits | Various diseases | China | China | Time Series Study | Meteorological factors |
| Outpatient visits | Various diseases | China | China | Retrospective time-series study | Air Pollutant Factors |

Table S7. Frequency table Across the Renal, cognitive and Urological characteristics.

| Response Variables | Primary Disease | Diseases | Region | Location | Study Plan | Exposure Variables |
| --- | --- | --- | --- | --- | --- | --- |
| Mortality | Renal and Urological Diseases | Renal disease | China | China | Time Series Study | Air Pollutant Factors |
| Outpatient visits | Renal and Urological Diseases | Urinary diseases | China | China | Machine learning model | Temperature /Humidity |
| Emergency room visits | Renal and Urological Diseases | Urolithiasis | Europe | Spain | Time Series Study | Temperature Factors |
| Hospital Admissions | Renal and Urological Diseases | Urolithiasis | China | China | Time Series Study | Temperature Factors |
| Hospital Admissions | Renal and Urological Diseases | Urolithiasis | China | China | Time Series Study | Temperature Factors |
| Hospital Admissions | Renal and Urological Diseases | Urolithiasis | China | China | Time Series Study | Temperature Factors |
| Hospital Admissions | Renal and Urological Diseases | Urolithiasis | China | China | Time Series Study | Temperature Factors |
| Hospitalisation | Renal and Urological Diseases | Urolithiasis | China | China | Time Series Study | Temperature Factors |
| Incidence | Cognitive disorders | Cognitive | China | China | Time Series Study | Temperature Factors |
| Emergency room visits | Cognitive disorders | Depression | Asian Countries | South Korea | Time Series Study | Meteorological Factors |
| Outpatient visits | Cognitive disorders | Depression | China | China | Time Series Study | Air Pollutant Factors |
| Death Outcomes | Cognitive disorders | Mental disorders | China | China | Case Crossover Study | Temperature Factors |
| Hospital Admissions | Cognitive disorders | Mental disorders | North America | USA | Cross-sectional study | Air Pollutant Factors |
| Hospital visits | Cognitive disorders | Mental disorders | China | China | Time Series Study | Temperature Factors |
| Hospitalisation | Cognitive disorders | Mental disorders | China | China | Others | Temperature Factors |
| Emergency room visits | Cognitive disorders | Mental disorders | North America | USA | Time Series Study | Meteorological Factors |
| Death Outcomes | Cognitive disorders | Suicide | China | China | Cohort Study | Air Pollutant Factors |
| Physiological Metric | Cognitive disorders | Suicide | Asian Countries | South Korea | Retrospective study | Temperature Factors |
| Incidence | Cognitive disorders | Suicide | Asian Countries | South Korea | Retrospective time-series study | Temperature /Humidity |
| Mortality | Cognitive disorders | Suicide | Asian Countries | Japan | Time Series Study | Temperature Factors |
| Incidence | Cognitive disorders | Trauma | China | China | Ecological study | Temperature Factors |

Table S8. Frequency table for Sampling design, Primary Diseases, and health outcomes.

| Characteristic | Case Crossover Study N = 36*^1^* | Cohort Study N = 22*^1^* | Cross-sectional study N = 13*^1^* | Deep learning N = 2*^1^* | Ecological study N = 11*^1^* | Machine learning model N = 3*^1^* | Observational study N = 18*^1^* | Others N = 24*^1^* | Retrospective study N = 1*^1^* | Retrospective time-series study N = 13*^1^* | Time-stratified case-crossover N = 10*^1^* | Time Series Study N = 136*^1^* |
| --- | --- | --- | --- | --- | --- | --- | --- | --- | --- | --- | --- | --- |
| Primary Diseases | | | | | | | | | | | | |
| Cancer | 0 (0%) | 2 (9.1%) | 1 (7.7%) | 0 (0%) | 1 (9.1%) | 0 (0%) | 0 (0%) | 3 (13%) | 0 (0%) | 0 (0%) | 0 (0%) | 2 (1.5%) |
| Cardiovascular | 8 (22%) | 6 (27%) | 2 (15%) | 0 (0%) | 1 (9.1%) | 1 (33%) | 7 (39%) | 3 (13%) | 1 (100%) | 1 (7.7%) | 3 (30%) | 28 (21%) |
| Cardiovascular and Respiratory | 3 (8.3%) | 0 (0%) | 0 (0%) | 0 (0%) | 1 (9.1%) | 0 (0%) | 1 (5.6%) | 0 (0%) | 0 (0%) | 0 (0%) | 0 (0%) | 6 (4.4%) |
| Dermatological | 0 (0%) | 0 (0%) | 0 (0%) | 0 (0%) | 1 (9.1%) | 0 (0%) | 1 (5.6%) | 0 (0%) | 0 (0%) | 0 (0%) | 0 (0%) | 0 (0%) |
| Infectious | 6 (17%) | 1 (4.5%) | 3 (23%) | 1 (50%) | 6 (55%) | 1 (33%) | 4 (22%) | 11 (46%) | 0 (0%) | 5 (38%) | 1 (10%) | 44 (32%) |
| Maternal and Fetal | 2 (5.6%) | 1 (4.5%) | 0 (0%) | 0 (0%) | 0 (0%) | 0 (0%) | 0 (0%) | 0 (0%) | 0 (0%) | 0 (0%) | 0 (0%) | 1 (0.7%) |
| Mental Health | 6 (17%) | 0 (0%) | 0 (0%) | 0 (0%) | 1 (9.1%) | 0 (0%) | 0 (0%) | 0 (0%) | 0 (0%) | 0 (0%) | 1 (10%) | 6 (4.4%) |
| Metabolic | 0 (0%) | 4 (18%) | 1 (7.7%) | 0 (0%) | 0 (0%) | 0 (0%) | 0 (0%) | 1 (4.2%) | 0 (0%) | 0 (0%) | 0 (0%) | 1 (0.7%) |
| Neurological | 2 (5.6%) | 0 (0%) | 1 (7.7%) | 0 (0%) | 0 (0%) | 0 (0%) | 0 (0%) | 2 (8.3%) | 0 (0%) | 0 (0%) | 2 (20%) | 4 (2.9%) |
| Renal | 2 (5.6%) | 0 (0%) | 0 (0%) | 0 (0%) | 0 (0%) | 0 (0%) | 0 (0%) | 1 (4.2%) | 0 (0%) | 0 (0%) | 0 (0%) | 8 (5.9%) |
| Respiratory | 7 (19%) | 8 (36%) | 5 (38%) | 1 (50%) | 0 (0%) | 1 (33%) | 5 (28%) | 3 (13%) | 0 (0%) | 7 (54%) | 3 (30%) | 36 (26%) |
| Primary Sampling Design | | | | | | | | | | | | |
| Birth Outcomes | 1 (2.8%) | 1 (4.5%) | 0 (0%) | 0 (0%) | 0 (0%) | 0 (0%) | 0 (0%) | 0 (0%) | 0 (0%) | 0 (0%) | 0 (0%) | 0 (0%) |
| Disease Outcome | 3 (8.3%) | 2 (9.1%) | 2 (15%) | 0 (0%) | 1 (9.1%) | 0 (0%) | 1 (5.6%) | 1 (4.2%) | 1 (100%) | 1 (7.7%) | 0 (0%) | 4 (2.9%) |
| Incidence/Prevalence | 9 (25%) | 6 (27%) | 2 (15%) | 0 (0%) | 4 (36%) | 1 (33%) | 6 (33%) | 6 (25%) | 0 (0%) | 3 (23%) | 1 (10%) | 32 (24%) |
| Infectious Outcomes | 2 (5.6%) | 0 (0%) | 2 (15%) | 1 (50%) | 2 (18%) | 0 (0%) | 1 (5.6%) | 6 (25%) | 0 (0%) | 5 (38%) | 0 (0%) | 12 (8.8%) |
| Morbidity | 8 (22%) | 8 (36%) | 4 (31%) | 1 (50%) | 4 (36%) | 0 (0%) | 8 (44%) | 2 (8.3%) | 0 (0%) | 3 (23%) | 4 (40%) | 68 (50%) |
| Mortality | 13 (36%) | 1 (4.5%) | 2 (15%) | 0 (0%) | 0 (0%) | 2 (67%) | 2 (11%) | 6 (25%) | 0 (0%) | 1 (7.7%) | 5 (50%) | 20 (15%) |
| Physiological Metric | 0 (0%) | 4 (18%) | 1 (7.7%) | 0 (0%) | 0 (0%) | 0 (0%) | 0 (0%) | 3 (13%) | 0 (0%) | 0 (0%) | 0 (0%) | 0 (0%) |
| *^1^*n (%) | | | | | | | | | | | | |
